# Prediction of radiogenic Sr and Pb isotope signatures in plants using diffusive gradients in thin films

**DOI:** 10.1007/s00216-026-06315-6

**Published:** 2026-02-02

**Authors:** Stefan Wagner, Jakob Santner, Markus Puschenreiter, Johanna Irrgeher, Thomas Prohaska

**Affiliations:** 1 Department General, Analytical and Physical Chemistry, Chair of General and Analytical Chemistry, Montanuniversität Leoben, Franz Josef-Straße 18, 8700 Leoben, Austria; 2 Institute of Plant Nutrition, Justus Liebig University Giessen, Heinrich-Buff-Ring 26-32, 35392 Giessen, Germany; 3 Department of Forest and Soil Sciences, Institute of Soil Research, BOKU University, Konrad-Lorenz-Straße 24, 3430 Tulln, Austria

**Keywords:** Passive sampling, Soil extraction, Metal stable isotopes, Geographical origin, MC-ICP-MS

## Abstract

This study evaluated the potential of the diffusive gradients in thin films (DGT) technique to assess radiogenic strontium (Sr) and lead (Pb) isotope signatures in bioavailable soil fractions as a proxy for plant uptake. Concentrations (*c*_DGT_) and isotope ratios of Sr (^87^Sr/^86^Sr) and Pb (^207^Pb/^206^Pb, ^208^Pb/^206^Pb, ^206^Pb/^204^Pb) assessed by DGT (TK100, Chelex), along with extractable (NH_4_NO_3_, NH_4_OAc, EDTA) and total Sr and Pb mass fractions and isotope ratios, were compared to those in *Lactuca sativa* L. (lettuce), *Triticum aestivum* L. (wheat), and *Raphanus sativus* L. (radish) grown on five geochemically distinct soils. Relative to conventional soil extraction, DGT significantly reduced matrix loads, facilitating isotope ratio measurements by multi-collector inductively coupled plasma mass spectrometry. DGT-labile Sr and Pb concentrations and isotope ratios reflected soil-specific geochemical signatures, allowing for clear differentiation among soils. Importantly, DGT-labile isotope ratios closely matched those in plant tissues across soils and species within analytical uncertainty, demonstrating that DGT captures the isotopically relevant bioavailable Sr and Pb pool without inducing significant mass-dependent isotopic fractionation. These findings establish DGT as a practical tool for bioavailable multi-isotope tracing with strong potential for applications in environmental forensics, food authentication, and archaeological provenance research.

**Graphical abstract:**

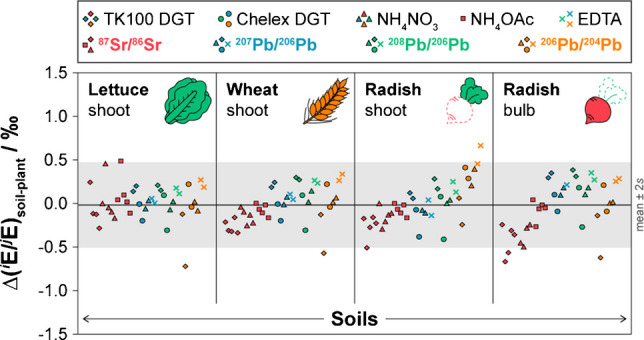

**Supplementary Information:**

The online version contains supplementary material available at 10.1007/s00216-026-06315-6.

## Introduction

Radiogenic isotope ratios of strontium (i.e., ^87^Sr/^86^Sr) and lead (e.g., ^207^Pb/^206^Pb, ^208^Pb/^206^Pb, ^206^Pb/^204^Pb) vary systematically across the Earth’s surface due to differences in bedrock age, geochemical evolution, and atmospheric deposition [1–3]. Initially applied in the geosciences [4, 5], radiogenic Sr and Pb isotope variations are now widely used to trace biological mobility, material provenance, and contaminant sources in applications ranging from archaeology and ecology to environmental forensics and food authentication [6–11]. In soils, Sr isotopes are particularly informative for quantifying weathering regimes and agricultural inputs from liming and irrigation [12–15], while Pb isotopes are sensitive tracers of anthropogenic pollution pathways and legacy contamination from industrial and mining activities [16–18]. The growing range of such applications has been mainly driven by advances in multi-collector inductively coupled plasma mass spectrometry (MC-ICP-MS), enabling high-precision isotope ratio measurements with high sample throughput [19].

To assign radiogenic Sr and Pb isotope signatures in biological samples to their geographic origin, they must be compared against local baselines that reflect the bioavailable isotopic composition of the environment [2]. Bioavailable soil fractions are increasingly used for this purpose [20–22], as they incorporate both the regional geogenic and atmospheric isotopic signatures [23, 24], while reducing complications introduced by species-specific uptake mechanisms, trophic mixing, and physiological or ecological variability associated with plant or animal proxies [14, 25, 26]. However, for such baselines to be reliable, the bioavailable soil pools must be characterized using methods that accurately assess the isotopic fractions of Sr and Pb accessible to plants.

Uptake of Sr and Pb in plants occurs primarily via root absorption of soluble, exchangeable, and weakly complexed species in the rhizosphere. Traditional chemical soil extraction methods using neutral salt solutions (e.g., NH_4_NO_3_, NH_4_OAc) or chelating agents (e.g., EDTA) are commonly applied to approximate these bioavailable pools [27–29]. For example, ^87^Sr/^86^Sr ratios in NH_4_NO_3_ soil extracts have been shown to correspond with those in plant tissues and plant-derived food products [22, 30–32]. However, such methods are operationally defined, vary in selectivity, and do not mimic the kinetic uptake mechanisms of plant roots [33]. Furthermore, they typically require large soil quantities (20–100 g), are prone to elevated blanks from multistep reagent-intensive workflows, and yield complex matrices rich in major cations (e.g., Na^+^, K^+^, Ca^2+^) and/or organic compounds (e.g., acetate, EDTA) that cause spectral and non-spectral interferences during isotope ratio measurements [19, 27–29]. These limitations highlight the need for alternative sampling approaches that more closely mimic kinetic root uptake, require less sample material, and generate cleaner, low-matrix solutions that can be either analyzed directly or are easy to process for isotopic analysis.

The diffusive gradients in thin films (DGT) technique is an established analytical tool for assessing the dynamic availability of elements in soils to plants [33–35]. Acting as an infinite sink, DGT induces diffusion of labile species from soil solution into the sampler and desorption from soil solid phases upon solute depletion, mimicking plant root uptake under diffusion-limited conditions [33, 36]. Moreover, the capability of selective DGT techniques for targeted analyte sampling effectively reduces matrix interferences, potentially enabling more straightforward isotope ratio analysis via MC-ICP-MS by reducing or eliminating the need for laborious matrix separation procedures [37, 38]. Despite DGT’s established role in assessing metal bioavailability, its application for isotope ratio analysis remains largely underexplored, with only a few studies assessing stable isotope ratios in labile soil fractions [37–41]. Thus, a gap remains in understanding whether DGT-labile Sr and Pb fractions carry isotopic signatures that are reliably mirrored in plant tissues, and whether this relationship holds across different plant species and soil types.

The present study addresses this question by evaluating if radiogenic Sr and Pb isotope signatures assessed by DGT reflect those taken up in plants. We evaluated two DGT techniques, the recently developed TK100 DGT [38] and the widely used Chelex DGT [42], alongside traditional extraction and digestion methods across six geochemically distinct soils in comparison to plants grown in selected soils under controlled conditions. Our hypotheses included the following: (1) The DGT techniques enable selective sampling of labile, bioavailable Sr and Pb and reduce analytical interferences; (2) DGT-labile isotope ratios reflect environmental sources consistent with traditional methods of soil analysis; and (3) isotope ratios in plants match those of the DGT-labile soil fraction, independent of plant species or tissue type.

## Materials and methods

To comply with notation conventions in DGT literature, the symbol *c* is used throughout this work for both molar and mass concentrations in solution instead of the designated symbols *c* and *γ* recommended by the International Union of Pure and Applied Chemistry (IUPAC). For isotope ratios and isotopic differences between two samples, the small delta (*δ*) and capital delta (*∆*) symbols are used, respectively. This notation expresses isotope ratios relative to an internationally accepted reference material, ensuring consistency and comparability of the reported data [43]. For completeness and further comparability, absolute isotope amount ratios are also provided. Details on general laboratory procedures, solutions, and materials are provided in the Supplementary Information (Text S1).

### Soil sampling and characterization

**Fig. 1:**
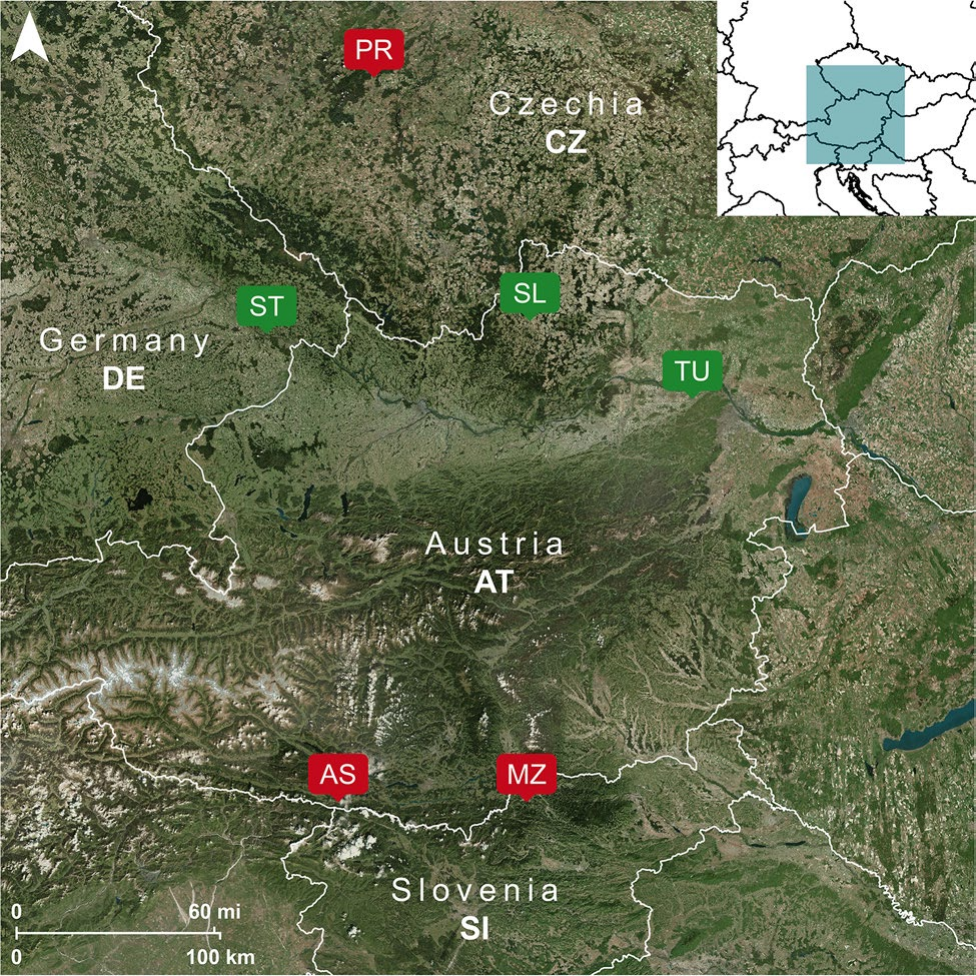
Geographical location of the soil sampling sites, with agricultural soils (TU, SL, ST) marked in green and contaminated soils (AS, MZ, PR) marked in red. Additional soil details are provided in the Supplementary Information (Table S1)

Six soil samples (~ 30–80 kg; 0–30 cm depth) were collected in 2018 from different sites in Austria (AT), Czechia (CZ), Germany (DE), and Slovenia (SI) (Fig. 1, Table S1). Soils TU, SL, and ST were collected from uncontaminated agricultural sites, whereas AS, MZ, and PR were collected from contaminated sites with elevated Pb levels due to historic Pb mining and smelting activities [44–46]. The soils were air-dried, sieved to either ≤ 2 mm for soil analyses or ≤ 4 mm for plant experiments, and stored in the dark at room temperature until use. Soils were digested according to Prohaska et al. [16]. Extractable Sr was determined using NH_4_NO_3_ (*c* = 1 mol L^−1^) and NH_4_OAc (*c* = 1 mol L^−1^, pH 7) extraction [27, 28]. Extractable Pb was determined using NH_4_NO_3_ (*c* = 1 mol L^−1^) and EDTA (*c* = 0.05 mol L^−1^) extraction [27, 29]. Soil digests and extracts were analyzed for Ca, Rb, Sr, and Pb using ICP-MS (measurement details are provided in section “Mass spectrometric analysis”). Both NH_4_OAc and EDTA extracts were subjected to microwave-assisted acid digestion to decompose dissolved organic compounds and the organic matrix that could interfere in matrix separation by column chromatography for subsequent isotope ratio measurements. Details of the total soil and extract digestion procedures are provided in the Supplementary Information (Text S2). Soil pH, CaCO_3_, organic C, clay, silt, and sand contents, cation exchange capacity (CEC), and water holding capacity (WHC) were determined following routine procedures [28, 47]. Geolithological and chronological information was retrieved from databases and literature references [44, 48].

### DGT application in soils

The TK100 DGT [38] and Chelex DGT [42] techniques were used to assess the capability of DGT as a proxy for Sr and Pb bioavailability and their radiogenic isotope signatures in plants. Both devices contained a binding layer, agarose cross-linked polyacrylamide (APA) diffusive gel (thickness 0.8 mm), and PES membrane (pore size 0.45 µm, thickness 0.15 mm). For TK100 DGTs, the binding layer was a TK100 membrane (thickness 0.7 mm; TrisKem International, FR), prepared as described in Wagner et al. [38]. For Chelex DGTs, the binding layer was a polyacrylamide gel (thickness 0.4 mm) containing milled Chelex-100 resin (particle size ≤ 10 µm) to provide a more homogeneous resin distribution while maintaining an adequate binding capacity compared to the original gel formulation [49]. Milling of the original Chelex-100 (sodium form, particle size 37–75 µm; Sigma-Aldrich, AT) was accomplished according to Doolette et al. [50]. Diffusive gels and PES membranes were preconditioned in NaNO_3_ (*c* = 0.01 mol L^−1^) for ≥ 24 h before assembly. For deployment, ~ 150 g of each soil was mixed with ultrapure water to reach 100% WHC, incubated at 24 °C for 24 h, and applied as a ~ 2-mm-thick layer (~ 2 g dry weight) on the DGT devices. The DGTs were gently tapped to ensure continuous soil contact and then placed in closed plastic boxes for 24 h at 24 °C in an incubator. Subsamples (*V* = ~ 20 mL) of the incubated soils were used to collect soil solution as described previously [38].

After deployment, DGTs were cleaned of soil and disassembled, and the binding layers were retrieved. TK100 membranes were eluted in three consecutive steps: first with 10 mL HCl (*c* = 0.01 mol L^−1^) for 1 h to desorb co-bound matrix elements, second with 10 mL HCl (*c* = 2 mol L^−1^) for 24 h to desorb Sr and partly Pb, and third with 10 mL HCl (*c* = 6 mol L^−1^) for 24 h to desorb residual Pb [38]. For isotope ratio analysis, the second fraction was used for Sr (*f*_e_ = 0.75), whereas for Pb, the second and third fractions were pooled to obtain adequate elution recovery (*f*_e_ = 0.87). Chelex gels were subjected to single-step elution in 10 mL HNO_3_ (*c* = 1 mol L^−1^) for 24 h, as quantitative elution of both Sr (*f*_e_ = 1.00) [51] and Pb (*f*_e_ = 0.95) [52] has been reported under these conditions. DGT-labile concentrations (*c*_DGT_) were calculated from the analyte mass bound to the binding layer (*m*_bl_) using Eqs. 1 and 2, and *R* values were calculated as the ratio of *c*_DGT_ values and soil solution concentrations (*c*_soln_) using Eq. 3:

1 $${m}_{\mathrm{bl}}=\frac{{c}_{\mathrm{e}}\times ({V}_{\mathrm{bl}}+{V}_{\mathrm{e}})}{{f}_{\mathrm{e}}}$$

2 $${c}_{\mathrm{DGT}}=\frac{{m}_{\mathrm{bl}}\times\Delta g}{D\;{\times \;A}_{\mathrm{p}}\times t}$$

3 $$R=\frac{c_\mathrm{DGT}}{c_\mathrm{soln}}$$

where *c*_e_ is the analyte mass concentration in the eluate, *V*_bl_ is the binding layer volume, *V*_e_ is the eluate volume, *f*_e_ is the elution recovery, Δ*g* is the diffusion layer thickness, *A*_p_ is the exposure window area, *D* is the diffusion coefficient, and *t* is the deployment time. For *f*_e_, the second eluate fraction was considered for Sr (*f*_e_ = 0.75) and the third fraction for Pb (*f*_e_ = 0.56). Diffusion coefficients for Sr and Pb at 24 °C were taken from Wagner et al. [38]. DGTs treated as samples but without soil contact were included as blanks. Background equivalent concentrations (BECs), limits of detection (*x*_L_), and limits of quantification (*x*_Q_) of the TK100 and Chelex DGT for Sr and Pb were consistent with previous reports [38] and are provided in the Supplementary Information (Table S2).

### Plant experiment and sample preparation

The experimental plant species were *Lactuca sativa* L. var. crispa (lettuce), *Triticum aestivum* L. cv. gr70 (wheat), and *Raphanus sativus* L. cv. Cherry Belle (radish). These plant species were chosen as representative crops to investigate the suitability of the DGT techniques and soil extraction methods for isotopic tracing of plant origin. Seeds were purchased from Vielfalt erleben GmbH (Vienna, AT) and Samen Maier GmbH (Taiskirchen im Innkreis, AT) and germinated for 5 days on moist tissue in Petri dishes. Four uniform seedlings per species were transplanted in 1.45-L pots filled with ~ 1200 g of each of the six experimental soils (bulk density ~ 1.1 g cm^−3^), amended with acid-washed quartz sand (*w* = 10%) to improve aeration and avoid reducing conditions that could affect metal speciation and lability as well as root development. Each species-soil combination had four replicates. Plant growth was conducted in a controlled greenhouse environment with average day/night temperatures of 21 °C/16 °C (14 h/10 h), relative humidity of 55–70%, and a minimum photosynthetic active radiation intensity of 300 µmol m^−2^ s^−1^. At the time of seedling transplantation, soils were fertilized with a mixed NH_4_NO_3_ and NH_4_H_2_PO_4_ solution to provide adequate amounts of N (*w* = 150 mg kg^−1^) and P (*w* = 133 mg kg^−1^) for plant growth. Soil moisture was maintained at 50–60% WHC by gravimetric irrigation twice per week using deionized water.

Plants were harvested 30–52 days after planting, depending on the development status and the formation of necrosis indicating toxicity when grown in the contaminated soils. Replicates of the same plant species were harvested at the same time. Plants were cut immediately above the soil surface and separated into belowground and aboveground tissues. For lettuce and wheat, only the aboveground tissues (shoots) were further processed, while radish included both shoots and fully developed bulbs. Details on plant sample preparation and digestion are provided in the Supplementary Information (Text S3).

Plant growth in the PR soil was severely inhibited by the high Pb mass fraction in this soil (*w*(Pb)_total_ = 5009 ± 113 mg kg^−1^; Table S1), which exceeds typical safety limits by more than one order of magnitude and falls well within the range known to impair root development and net photosynthesis [53]. Therefore, no plant data could be collected for this soil.

### Matrix separation

To obtain purified Sr and Pb fractions for isotope ratio analysis, soil extracts, soil digests, and plant digests were processed using an automated Sr-Pb/matrix separation method based on DGA resin (TrisKem International, FR) and the prepFAST MC system (Elemental Scientific, USA) [54, 55]. Sr eluates from TK100 DGTs were subjected to manual column separation using the Sr spec resin (TrisKem International, FR) [56], while Pb eluates of TK100 and Chelex DGTs were analyzed without additional purification. Procedural details are provided in the Supplementary Information (Text S4, Table S3).

### Mass spectrometric analysis

Elemental analysis was performed using quadrupole ICP-MS (Elan 9000 DRCe, Perkin Elmer, USA) and sector-field ICP-MS (Element XR, Thermo Fisher Scientific, USA) with internal normalization and external calibration as described previously [38]. Isotope ratio analysis of Sr and Pb was performed using MC-ICP-MS (Nu Sapphire or Nu Plasma HR, Nu Instruments, UK) coupled to a membrane desolvator (Aridus II, Teledyne CETAC, USA; or Apex Omega, ESI, USA). Optimization procedures and instrumental parameters of all MC-ICP-MS measurements are provided in the Supplementary Information (Text S5, Table S4). Correction of blank levels and residual isobaric interferences was accomplished as reported previously [54, 57]. To account for instrumental isotopic fractionation (IIF) and potential natural mass-dependent fractionation (MDF), ^87^Sr/^86^Sr was corrected internally using a fixed ^88^Sr/^86^Sr ratio of 8.37861 from the SRM 987 certificate [57]. IIF of ^207^Pb/^206^Pb, ^208^Pb/^206^Pb, and ^206^Pb/^204^Pb was corrected by standard-sample bracketing (SSB) [54]. Measurements were performed in a SSB sequence at Sr and Pb concentrations in samples and bracketing standards (matched at ± 10%) of 10–50 µg L^−1^ and 1.5–25 µg L^−1^, respectively, in HNO_3_ (*w* = 2%) for digests, extracts, and Chelex DGT eluates, or HCl (*w* = 2%) for TK100 DGT eluates. Isotope ratios and isotopic differences are reported as *δ*- and *∆*-values (both in ‰), respectively, according to Eqs. 4 and 5:

4 $${\delta }_{\mathrm{std}}{(^i\mathrm{E}/^j\mathrm{E})}_{\mathrm{spl}}=\frac{{R{^\prime}}_{\!\mathrm{spl}}}{{R{^\prime}}_{\!\mathrm{std}}}-1$$

5 $$\Delta {(^i\mathrm{E}/^j\mathrm{E})}_{\mathrm{spl}1-\mathrm{spl}2}={{\delta }_{\mathrm{std}}(^i\mathrm{E}/^j\mathrm{E})}_{\mathrm{spl}1}-{{\delta }_{\mathrm{std}}(^i\mathrm{E}/^j\mathrm{E})}_{\mathrm{spl}2}$$

where ^*i*^E/^*j*^E refers to ^87^Sr/^86^Sr, ^207^Pb/^206^Pb, ^208^Pb/^206^Pb, or ^206^Pb/^204^Pb and *R’* is the blank-, interference-, and IIF-corrected isotope amount ratio *n*(^*i*^E)/*n*(^*j*^E) of the sample (spl) or isotopic standard (std). Because the same isotopic standards were used in all Sr (SRM 987, NIST, USA) and Pb (SRM 981, NIST, USA) isotope ratio analyses, the index std is omitted for simplicity. Absolute Sr and Pb isotope amount ratios are provided in the Supplementary Information.

### Analytical performance

The analytical performance of elemental and isotope ratio analysis was assessed using certified reference materials (CRMs) and in-house quality controls (QCs). For elemental analyses, QCs were prepared by mixing certified single- and multi-element solutions and measured every 6–12 samples [38], yielding recoveries within ± 10% of target values across runs. To validate elemental quantification in digests, soil (ISE 885; WEPAL, NL) and plant (SRM 1547, NIST; IPE 200, WEPAL) CRMs were included, resulting in quantitative Sr and Pb recoveries. Isotope ratio accuracy was verified through repeated analysis of isotopic CRMs (SRM 987, SRM 981), providing *δ*-values covering the certified value (*δ* = 0‰) within the measurement uncertainty. Matrix separation efficiency and potential on-column isotopic fractionation were evaluated by processing spiked SRM 987 and SRM 981 solutions under representative sample conditions in each separation batch, yielding *δ*-values between −0.19 and 0.14‰. Measured ^87^Sr/^86^Sr and ^207^Pb/^206^Pb ratios of SRM 1547 agreed with literature values [22, 58], while for ISE 885 and IPE 200, these values are reported here for the first time. Full validation data are provided in the Supplementary Information (Table S5, S6).

### Uncertainty calculation and statistical analysis

Expanded uncertainties (*U*, *k* = 2) of isotope ratios were calculated using a Kragten approach [57, 59] and included both the combined measurement uncertainty and repeatability between sample replicates [38]. For reporting *U* (*k* = 2) on *∆*-values, *U* (*k* = 2) of both soil and plant samples were combined. Statistical differences in elemental data were assessed by Welch’s ANOVA (one-way) followed by Games-Howell post hoc test due to unequal variances using Jamovi (version 2.3.28). Statistical differences between two mean values of relative isotope ratio data were assessed considering their *U* (*k* = 2) [37, 60]. Linear regressions were computed and visualized using GraphPad Prism (GraphPad Software, USA). Unless stated otherwise, elemental concentrations, mass fractions, and their ratios are reported as means ± standard deviations (*s*), and isotope ratios as means ± *U* (*k* = 2).

## Results and discussion

### Soil properties

The six soils covered a range of geographical, geological, and physicochemical conditions, reflecting diverse land use histories, parent materials, and soil types across Central Europe (Table S1). Total Sr mass fractions ranged from 52.0 (PR) to 163 mg kg^−1^ (SL), consistent with global background values [61]. Extractable Sr, assessed by NH_4_NO_3_ and NH_4_OAc, accounted for 2–7% of total Sr. NH_4_NO_3_-extractable Sr was 8–16% lower (SL, ST, MZ; *p* < 0.05), 18% higher (PR; *p* < 0.001), or not significantly different (TU, AS; *p* > 0.05) than NH_4_OAc-extractable Sr. These variable differences indicate that Sr extractability from exchangeable pools is strongly soil-dependent, likely reflecting differences in mineral composition and surface reactivity between the soils. Mass fraction ratios of Rb/Sr in total soil fractions varied from 0.57 (MZ) to 2.20 (ST), reflecting distinct lithological backgrounds [62].

Total Pb mass fractions ranged from 27.6 to 45.9 mg kg^−1^ in the uncontaminated agricultural soils TU, SL, and ST, up to 1040 to 5009 mg kg^−1^ in the contaminated soils AS, MZ, and PR. Thus, the three contaminated soils had Pb mass fractions more than ten times above the Austrian guideline limit of 100 mg kg^−1^ for Pb in agricultural soils [63]. However, NH_4_NO_3_-extractable Pb in AS, MZ, and PR was low, varying from 0.50 to 12.8 mg kg^−1^ and constituting < 1% of total Pb. EDTA-extractable Pb mirrored the total Pb pattern, ranging from 4.04 to 2810 mg kg^−1^ and accounting for 14–66% of total Pb. Extractability of Pb by EDTA was significantly higher (*p* < 0.001) in contaminated soils (61.9 ± 4.7%, *n* = 9) than in uncontaminated soils (21.8 ± 11.6%, *n* = 9), reflecting the greater lability and potential bioavailability of Pb with increasing total Pb in soil [64].

The soils also varied substantially in pH, including four acidic soils (SL, ST, AS, PR) with pH between 4.5 and 5.0, and two soils (TU, MZ) with close to neutral pH, varying from 7.0 to 7.3, which also contained significant amounts of CaCO_3_ between 83 and 115 g kg^−1^. Soil organic carbon ranged from 9.85 g kg^−1^ in ST to 67.1 g kg^−1^ in MZ. Cation exchange capacity ranged from 51 mmol_c_ kg^−1^ in PR to 340 mmol_c_ kg^−1^ in TU, generally aligning with clay content, which ranged from 82 (PR, MZ) to 268 g kg^−1^ (TU).

### DGT concentrations of Sr and Pb

DGT concentrations (*c*_DGT_) of Sr and Pb in the six soils differed markedly between the two applied DGT techniques, with consistently higher *c*_DGT_ and *R* values for both Sr and Pb determined by TK100 DGT compared to Chelex DGT (Table 1). This difference was particularly striking for Sr, where *c*_DGT_(Sr) values ranged from 42.9 to 174 µg L^−1^ for TK100 DGT but remained ≤ 10 µg L^−1^ for Chelex DGT across all soils. The observed difference was most pronounced for the calcareous soils TU and MZ, where *c*_DGT_(Sr) was 23 and 30 times higher for TK100 DGT than for Chelex DGT, respectively. This finding is consistent with nonquantitative uptake of Sr by Chelex DGT due to competitive effects between Sr^2+^ and other divalent metal cations with either higher selectivity for the iminodiacetate groups in the Chelex resin (e.g., Cu^2+^, Pb^2+^, Zn^2+^) [65] or higher relative abundance in labile element fractions in the soil (e.g., Ca^2+^, Mg^2+^) [66]. Accordingly, the weakly adsorbed Sr^2+^ on the Chelex binding layer was replaced via ion exchange during sampling, resulting in a significant underestimation of labile Sr by Chelex DGT, especially under the highly competitive conditions of calcareous soils. The high selectivity and capacity of the TK100 DGT for Sr^2+^ effectively overcomes this limitation, allowing for quantitative analysis of labile Sr across different soil types.

**Table 1:** DGT concentrations (*c*_DGT_) and *R* values (*c*_DGT_/*c*_soln_) of Sr and Pb in soils^a^

**Soil**	**Sr**				**Pb**			
	**TK100 DGT**		**Chelex DGT**		**TK100 DGT**		**Chelex DGT**	
	***c***_**DGT **_**/ (µg L**^**−1**^)	***R***	***c***_**DGT **_**/ (µg L**^**−1**^)	***R***	***c***_**DGT **_**/ (µg L**^**−1**^)	***R***	***c***_**DGT **_**/ (µg L**^**−1**^)	***R***
TU	174 ± 23	0.66 ± 0.07	7.58 ± 1.01	0.03 ± 0.01	< *x*_L_	n.a	< *x*_L_	n.a
SL	57.8 ± 2.4	0.32 ± 0.04	10.5 ± 0.2	0.06 ± 0.01	0.95	1.00	0.19	0.20
ST	42.9 ± 2.4	0.77 ± 0.01	6.65 ± 0.68	0.12 ± 0.01	< *x*_L_	n.a	< *x*_L_	n.a
AS	46.6 ± 3.0	0.21 ± 0.04	8.90 ± 0.26	0.04 ± 0.01	231 ± 13	0.64 ± 0.05	77.3 ± 5.2	0.21 ± 0.01
MZ	168 ± 22	0.19 ± 0.03	5.80 ± 0.73	0.01 ± < 0.01	74.8 ± 6.8	0.69 ± 0.05	26.1 ± 2.7	0.24 ± 0.02
PR	52.2 ± 3.2	0.34 ± 0.02	7.62 ± 0.35	0.05 ± < 0.01	575 ± 42	1.66 ± 0.16	56.0 ± 1.3	0.16 ± 0.01

^a^Values are means ± *s* (*n* = 3). TK100 DGT data for soils SL, AS, and MZ was taken from Wagner et al. [38]. No errors are shown for values < *x*_Q_. *n.a* not analyzed

For Pb, DGT concentrations in the agricultural soils TU and ST were below the limit of detection (*x*_L_) and in SL below the limit of quantification (*x*_Q_) (Table S2), which is consistent with the ultra-low levels of Pb in soil solutions typically found under uncontaminated conditions [66]. In the contaminated soils AS, MZ, and PR, DGT-labile Pb was high, with *c*_DGT_(Pb) values ranging from 74.8 to 575 µg L^−1^ for TK100 DGT and 26.1 to 77.3 µg L^−1^ for Chelex DGT (Table 1). Thus, as for Sr, DGT-labile Pb was significantly higher when assessed with TK100 DGT compared to Chelex DGT, reaching up to ten times higher *c*_DGT_(Pb) values in the highly contaminated PR soil. Despite the similar results for Sr and Pb, competitive effects are unlikely to explain this observation for Pb, given the high selectivity of Chelex for Pb^2+^ [67]. In a previous laboratory experiment deploying TK100 DGTs (*n* = 3) for 24 h in unbuffered media (*V* = 2 L), we found a decrease in solution pH from ~ 5 to ~ 4 during the deployment period [38], suggesting that the TK100 DGT affects pH via proton exchange at the di(2-ethyl-hexyl)phosphoric acid phase of the TK100 resin. Depending on the soil buffering capacity, this mechanism may locally acidify the soil during sampling, resulting in enhanced Pb resupply from the soil solid phase into the soil solution due to the pH-dependent release of sorbed Pb [68]. Consequently, significantly higher *R* values were obtained for Pb assessed by TK100 DGT compared to Chelex DGT (Table 1), even exceeding a value of 1 for the weakly buffered PR soil, demonstrating Pb mobilization by the TK100 DGT. These findings indicate that proton exchange at the TK100 binding phase can modify metal resupply kinetics via altered acid-base equilibria at the sampling interface, resulting in *c*_DGT_ values that may deviate from those obtained under strictly passive sampling conditions. The magnitude of this effect can, however, be quantified using speciation models and analytical expressions [69, 70], allowing its influence on metal lability to be constrained rather than treated as an uncontrolled artifact. Auspiciously, this effect also offers a unique opportunity to probe environmentally relevant mobilization mechanisms, as comparable microscale acidification is well documented in soils due to root activity and microbially mediated proton or organic acid exudation [71–73]. Conversely, in environmental systems with large and well-mixed aqueous reservoirs, such as rivers, any proton release from the binding phase is expected to be rapidly dissipated by advective transport, such that TK100 DGT effectively operates as a purely passive sampler.

### Plant uptake of Sr and Pb in relation to soil bioavailability

The mass uptake of Sr and Pb in plant tissues varied significantly between soils, plant species, and plant parts (Table S7). Across all soils (*n* = 6), maximum Sr mass fractions were found in radish shoots (82.2 ± 8.8 mg kg^−1^) followed by lettuce shoots (29.8 ± 7.2 mg kg^−1^), radish bulbs (25.2 ± 10.2 mg kg^−1^), and wheat shoots (20.7 ± 11.7 mg kg^−1^). For Pb, tissue mass fractions in uncontaminated soils (*n* = 3) followed: lettuce shoots (0.25 ± 0.27 mg kg^−1^) > radish bulbs (0.16 ± 0.06 mg kg^−1^) > radish shoots (0.11 ± 0.01 mg kg^−1^) > wheat shoots (0.08 ± 0.04 mg kg^−1^), while in contaminated soils (*n* = 3) the trend was: radish bulbs (236 ± 281 mg kg^−1^) > lettuce shoots (23.6 ± 21.6 mg kg^−1^) > radish shoots (18.4 ± 11.6 mg kg^−1^) > wheat shoots (15.9 ± 14.5 mg kg^−1^). These values generally agree with reported literature ranges for these plant species grown in soils with comparable total Sr and Pb mass fractions (Table S1) [61, 74, 75].

The uptake of Sr was soil-dependent for all plant species (*p* < 0.01) except radish shoots (*p* > 0.05) (Table S7). However, Sr uptake did not correlate with the TK100 DGT-labile or NH_4_NO_3_- and NH_4_OAc-extractable fractions (*p* > 0.05) (Table S8). This lack of correlation is consistent with the understanding that Sr uptake follows that of Ca and is primarily controlled by mass flow [76–78], a process driven by transpiration-induced water flux. Under such conditions, the elemental concentration in the soil solution and the rate of water uptake are more critical than the size of the labile or exchangeable pool [79]. Thus, for elements such as Sr, whose uptake is driven by mass flow and subject to complex ion competition, methods such as DGT may have limited predictive power for elemental mass uptake in plants because they do not mechanistically mimic root uptake under mass flow-controlled conditions [33].

For Pb, plant uptake was also soil-dependent (*p* < 0.01), with significantly elevated mass fractions in plants grown in the contaminated soils AS and MZ (Table S7). Noteworthy, Pb mass fractions in all plants grown on these soils clearly exceeded the maximum level of 0.1 mg kg^−1^ (wet weight) for Pb in most edible plant parts set by the European Commission [80], considering a conservative water content estimate of 95% [81], highlighting the risk posed by legacy contamination. Although not significant (*p* > 0.05), the consistently higher Pb mass fractions in radish bulbs compared to shoots are confirmed by previous studies [74, 82] and indicate restricted Pb translocation from belowground to aboveground tissues, possibly as a mechanism to cope with Pb toxicity via sequestration in rhizodermal or cortical cells [83].

Maximum Pb mass fractions were consistently observed in plants grown on AS, despite the two times lower total Pb mass fraction in AS compared to MZ (Table S1). This is consistent with the lower pH and CEC of AS compared to MZ and confirms previous work showing that Pb bioavailability is primarily controlled by its solubility rather than its total content in soil [61, 84]. The bioavailability indices differed considerably in their ability to indicate this soil dependency of Pb availability. EDTA extraction did not indicate this difference in bioavailability but rather reflected the total Pb content, with about two times lower EDTA-extractable Pb in AS than MZ. Conversely, NH_4_NO_3_ extraction overestimated Pb bioavailability by almost an order of magnitude (Table S1). In contrast, labile Pb assessed by the two DGT techniques corresponded well with the observed differences in plant uptake, as evidenced by similar ratios of *c*_DGT_(Pb) and plant shoot *w*(Pb) between AS and MZ (TK100 DGT = 3.1 ± 0.2, Chelex DGT = 3.0 ± 0.5, plant shoot average = 4.0 ± 1.2). This confirms that DGT samples the Pb pool most relevant for plant uptake, providing a more robust and predictive indicator of Pb bioavailability than the applied extraction methods.

### Matrix composition of DGT eluates compared to soil extracts

A potential advantage of DGT for isotope ratio analysis is its ability to selectively accumulate target analytes while reducing matrix interferences. To evaluate this, DGT eluates and soil extracts were analyzed for Ca and Rb, which are the primary matrix elements interfering with accurate Sr isotope ratio measurements in bioavailable soil fractions [30, 38, 54]. For Pb, elevated Ca can suppress ICP-MS signals and compromise the efficiency of column-based purification procedures [55]. Because Pb concentrations in DGT eluates of uncontaminated soils were < *x*_Q_ (Table S2), only samples of the contaminated soils (AS, MZ, PR) were considered for Pb in this evaluation.

**Fig. 2:**
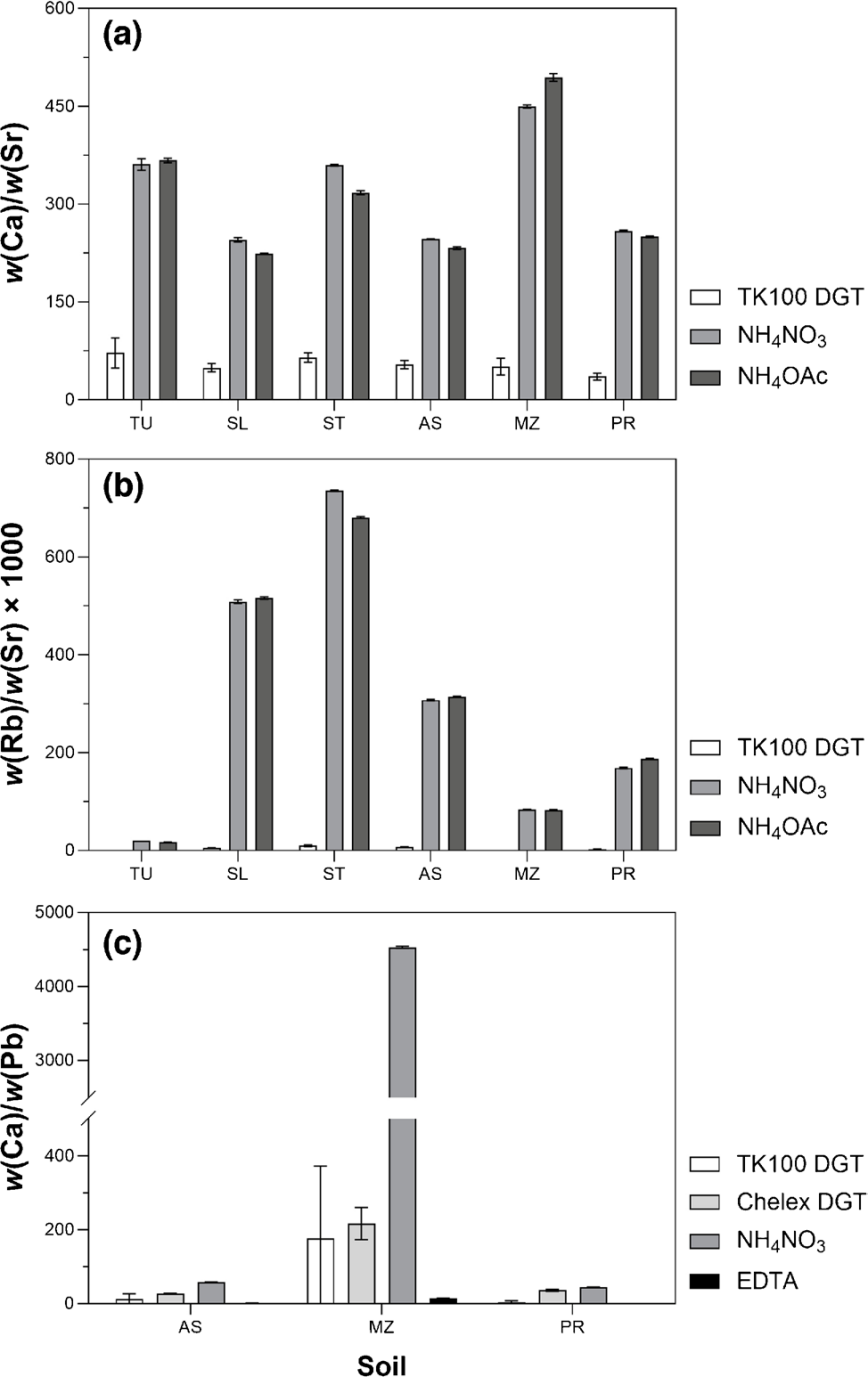
Mass fraction (*w*) ratios of Ca/Sr (**a**), Rb/Sr × 1000 (**b**), and Ca/Pb (**c**) in the DGT eluates and soil extracts of the three uncontaminated agricultural soils (TU, SL, ST) and three contaminated soils (AS, MZ, PR). Values are means ± *s* (*n* = 3). Exact values are provided in the Supplementary Information (Table S9)

As shown in Fig. 2, TK100 DGT eluates had the lowest overall matrix load, demonstrating effective matrix reduction via selective sampling and three-step elution. Across all soils (*n* = 6), mean Ca/Sr mass fraction ratios in TK100 eluates (54.4 ± 12.6) were significantly lower than those in NH_4_NO_3_ (320 ± 83) and NH_4_OAc extracts (314 ± 104) (*p* < 0.001), reflecting discrimination of the Ca matrix (Fig. 2a). Likewise, Rb/Sr × 1000 ratios were significantly lower in TK100 eluates (4.12 ± 3.82) than in NH_4_NO_3_ (304 ± 274) and NH_4_OAc extracts (300 ± 258) (*p* < 0.001), showing reduction of the isobaric Rb interference (Fig. 2b). The Chelex DGT was excluded from this comparison due to nonquantitative Sr uptake (Table 1).

For Pb, a similar pattern was observed (Fig. 2c). Across the contaminated soils AS, MZ, and PR (*n* = 3), mean Ca/Pb ratios in pooled (fractions 2 and 3) TK100 eluates (64.3 ± 97.1) were lower than those in Chelex eluates (93.5 ± 106.8) and especially NH_4_NO_3_ extracts (1545 ± 2587). EDTA extracts provided the lowest Ca/Pb ratios (5.27 ± 7.98), consistent with its strong Pb chelation (log *K*_Pb-EDTA_ ≈ 18) and efficient displacement of matrix cations [85]. However, while EDTA provides high extraction efficiency, it introduces a concentrated organic matrix that may interfere with chromatographic separation and requires complete ligand degradation to enable Pb isolation. In contrast, DGT eluates provide a simple inorganic acid matrix, offering enhanced analytical compatibility with column chemistry and MC-ICP-MS analysis.

### Sr and Pb isotope ratios in DGT-labile, extractable, and total soil fractions

**Fig. 3:**
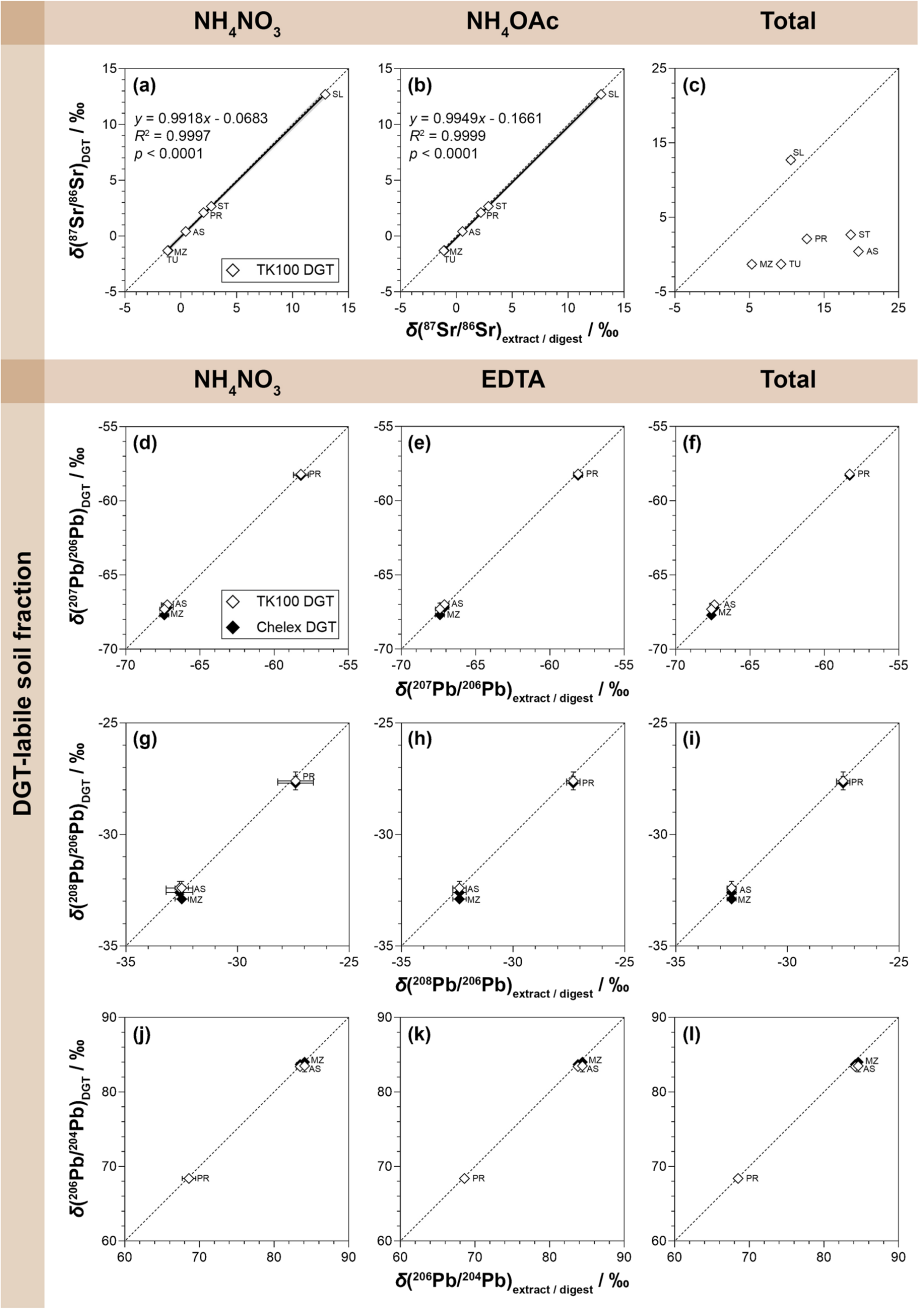
Relationship between Sr and Pb isotope ratios in DGT-labile soil fractions (*y*-axes) and those in extractable and total soil fractions (*x*-axes), including NH_4_NO_3_ and NH_4_OAc extracts and total digests for *δ*(^87^Sr/^86^Sr) (**a**–**c**), and NH_4_NO_3_ and EDTA extracts and total digests for *δ*(^207^Pb/^206^Pb) (**d–f**), *δ*(^208^Pb/^206^Pb) (**g–i**), and *δ*(^206^Pb/^204^Pb) (**j–l**). Values are means (*n* = 3) ± *U* (*k* = 2). Dashed lines indicate the 1:1 relationship (= line of identity). Solid lines and grey areas in **a** and **b** indicate the linear regression curve and 95% confidence intervals, respectively. Exact *δ*-values and absolute Sr and Pb isotope ratios of soils are provided in the Supplementary Information (Table S10, S11)

The relationship between DGT-labile, extractable, and total soil Sr and Pb isotope ratios was evaluated to assess whether DGT provides representative isotope signatures of bioavailable Sr and Pb. As shown in Fig. 3 and Table S10, the Sr and Pb isotope ratios in DGT-labile soil fractions varied significantly, with *δ*(^87^Sr/^86^Sr)_DGT_ ranging from −1.51 to 12.6‰ across all soils, and *δ*(^207^Pb/^206^Pb)_DGT_ from −67.3 to −58.2‰, *δ*(^208^Pb/^206^Pb)_DGT_ from −32.4 to −27.6‰, and *δ*(^206^Pb/^204^Pb)_DGT_ from 68.4 to 83.5‰ across contaminated soils (AS, MZ, PR). These values closely matched those in extractable soil fractions. For Sr, strong correlations were obtained for *δ*(^87^Sr/^86^Sr) between TK100 DGT and both NH_4_NO_3_ and NH_4_OAc (*R*^2^ > 0.999; Fig. 3a, b), without significant differences between these fractions (Table S10). For Pb, all isotope ratios overlapped with the corresponding line of identity (Fig. 3d–i), showing overall no significant differences between TK100 and Chelex DGT (Table S10), thus confirming that Pb mobilization by the TK100 DGT (Table 1) did not cause significant isotopic fractionation. These results indicate that the applied DGT techniques and conventional soil extraction methods sample similar radiogenic Sr and Pb isotope pools, ensuring data comparability.

In contrast, significant differences between DGT-labile/extractable and total soil fractions were observed consistently for *δ*(^87^Sr/^86^Sr) across all soils (Fig. 3c, Table S10), but not for *δ*(^207^Pb/^206^Pb) (Fig. 3f, Table S10), and only partially for *δ*(^208^Pb/^206^Pb) (Fig. 3i, Table S10) and *δ*(^206^Pb/^204^Pb) across contaminated soils (Fig. 3l, Table S10). This agrees with findings from previous studies [7, 30, 31, 86, 87] and reflects the differential contribution of Sr and Pb incorporated in isotopically distinct, mineral-bound pools. For Sr, the apparent differences clearly show the presence of these insoluble pools across all soils, which are not accessed by TK100 DGT or soil extraction. Interestingly, soil SL was the only case where the total soil fraction had significantly lower *δ*(^87^Sr/^86^Sr) than its DGT-labile and extractable counterparts. For the remaining soils, total fractions had significantly higher *δ*(^87^Sr/^86^Sr), suggesting variable, soil-dependent isotopic partitioning. These patterns may be attributed to differential mineral weathering in relation to pedogenetic processes, leading to varying isotopic differences in labile versus bulk pools, which become less distinct with increasing radiogenic contribution [20]. For Pb, little isotopic heterogeneity across fractions in contaminated soils suggests that the contribution of the mineral-bound pool is negligible compared to the labile pool, thereby minimizing isotopic partitioning [7, 87, 88]. In contrast, in uncontaminated soils, total fractions showed lower *δ*(^207^Pb/^206^Pb) and *δ*(^208^Pb/^206^Pb) and higher *δ*(^206^Pb/^204^Pb) than EDTA-extractable fractions, which were consistently significant for *δ*(^207^Pb/^206^Pb) and *δ*(^206^Pb/^204^Pb) and partially significant for *δ*(^208^Pb/^206^Pb). This pattern points to a stronger influence of Pb incorporated in the silicate fraction on the isotopic composition of the bulk pool under uncontaminated conditions [88].

The observed isotopic variability reflected differences in the lithological backgrounds and histories of the sampling sites (Table S1). For example, the relatively high *δ*(^87^Sr/^86^Sr) in soil SL reflected weathering of the Carboniferous granite bedrock [38], while the elevated *δ*(^87^Sr/^86^Sr) in ST can be attributed to the influence of Rb-bearing minerals such as biotite and muscovite originating from the underlying mica-schist and paragneiss. In comparison, soils TU and MZ showed low *δ*(^87^Sr/^86^Sr) values due to carbonate- and siliciclastic-rich alluvial parent materials with low Rb/Sr ratios, as confirmed by their total soil composition (Table S1). The Pb isotope ratios of the contaminated soils AS and MZ were nearly identical across all methods (Table S10), reflecting a common geological influence linked to the extensive carbonate-hosted Pb-Zn mineralization prevalent in both regions [38, 89]. This deposit is characterized by a homogeneous Pb isotope composition [90], which appears to dominate the bioavailable Pb isotope signature of local soils. In contrast, soil PR had significantly higher *δ*(^207^Pb/^206^Pb) and *δ*(^208^Pb/^206^Pb) and lower *δ*(^206^Pb/^204^Pb) values (Table S10), reflecting vein-type mineralization and distinct anthropogenic inputs from historical Pb mining and smelting [17, 91, 92]. Despite the similarity in Pb isotope ratios between soils AS and MZ, their *δ*(^87^Sr/^86^Sr) values differed significantly (Table S10), enabling effective differentiation. This highlights the strength of a multi-isotope approach, where radiogenic Sr and Pb isotope signatures provide complementary information. Notably, both systems can be assessed simultaneously from the same sample using the TK100 DGT.

### Relationship between DGT and plant Sr and Pb isotope ratios

**Fig. 4:**
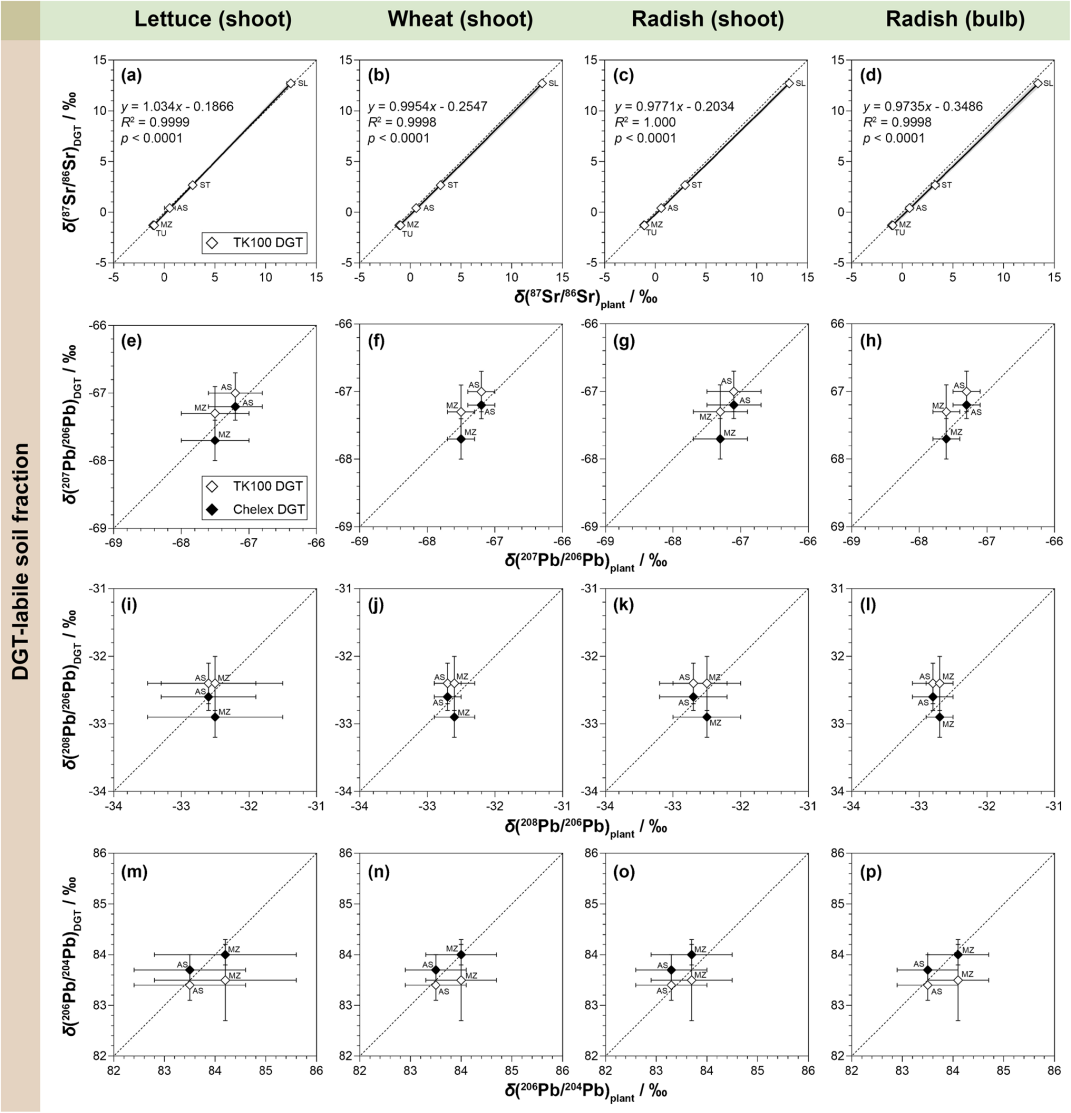
Relationship between Sr and Pb isotope ratios in DGT-labile soil fractions (*y*-axes) and those in plants (*x*-axes), including lettuce (shoot), wheat (shoot), and radish (shoot and bulb), for *δ*(^87^Sr/^86^Sr) (**a**–**d**), *δ*(^207^Pb/^206^Pb) (**e**–**h**), *δ*(^208^Pb/^206^Pb) (**i**–**l**), and *δ*(^206^Pb/^204^Pb) (**m**–**p**). Values are means (*n* = 3 for DGT, *n* = 4 for plants) ± *U* (*k* = 2). Dashed lines indicate the 1:1 relationship (= line of identity). Solid lines and grey areas in **a**–**d** indicate the linear regression curve and 95% confidence intervals, respectively. Exact *δ*-values and absolute Sr and Pb isotope ratios of plants are provided in the Supplementary Information (Table S12, S13)

To validate DGT as a proxy for radiogenic Sr and Pb isotope ratios in plants, it must be demonstrated that the DGT-labile pool accurately represents the isotopic composition of the fraction taken up by plants. If this relationship holds across different soil types and plant species, DGT can serve as a species-independent predictor of Sr and Pb isotope signatures in plants, eliminating the need for site-specific plant cultivation and analysis. As shown in Fig. 4, Sr and Pb isotope ratios in plant tissues closely matched those of the DGT-labile soil fractions, confirming that DGT provides a robust approach for predicting the radiogenic Sr and Pb isotope composition of plants across diverse soil-plant systems.

**Fig. 5:**
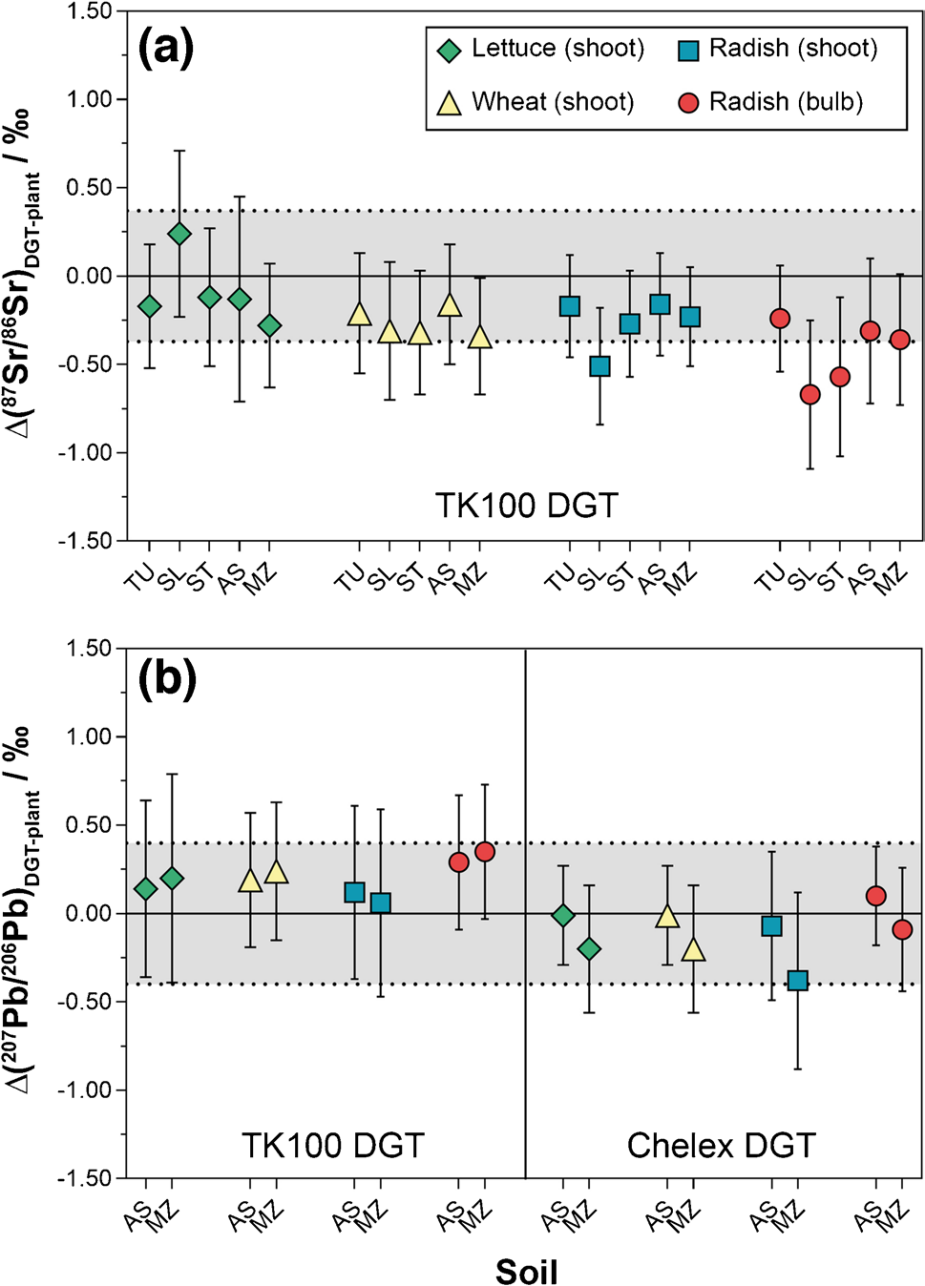
Isotopic differences, *∆*(^87^Sr/^86^Sr)_DGT-plant_ (**a**) and *∆*(^207^Pb/^206^Pb)_DGT-plant_ (**b**), between TK100 and/or Chelex DGT-labile soil fractions and plant tissues. Values are means ± *U* (*k* = 2). Grey areas indicate the average *U* (*k* = 2) of the shown *∆*-values. Exact *∆*-values, also including *∆*(^208^Pb/^206^Pb)_DGT-plant_ and *∆*(^206^Pb/^204^Pb)_DGT-plant_, are provided in the Supplementary Information (Table S14)

For Sr, *δ*(^87^Sr/^86^Sr) values in plants ranged from −1.12 to 13.4‰, with generally no significant differences between plant species or plant parts grown on the same soil (Table S12). Plant *δ*(^87^Sr/^86^Sr) values correlated strongly (*p* < 0.0001) with the TK100 DGT-labile soil fractions across all soils and plant species, as shown by *R*^2^ values > 0.999 and slopes close to 1 (Fig. 4a–d). This strong isotopic correlation contrasts with the lack of correlation between Sr mass fractions in plants and those in DGT-labile or extractable soil fractions (Table S8), indicating that, while Sr uptake is strongly influenced by soil physicochemical properties and plant physiological factors [78], the ^87^Sr/^86^Sr ratio remains a passive tracer of the bioavailable pool’s geochemical signature and is independent of the uptake magnitude. The close agreement in *δ*(^87^Sr/^86^Sr) between plant and TK100 DGT was also confirmed by the low isotopic difference (*∆*(^87^Sr/^86^Sr)_DGT-plant_ = −0.26 ± 0.38‰; mean ± 2*s*, *n* = 20) across all soil-plant combinations (Fig. 5a, Table S14). Consequently, the radiogenic Sr isotope composition of plants was determined predominantly by the radiogenic isotopic signature of the labile Sr pool sampled by the TK100 DGT. Potential natural MDF, as previously reported for Sr uptake and translocation in plants [20], or diffusion, resin binding, and elution in different DGT methods [40, 93, 94], is further accounted for by internal correction of the ^87^Sr/^86^Sr ratio [57], providing an accurate reflection of the radiogenic isotope variations causing biogeochemical ^87^Sr/^86^Sr variability [21].

For Pb, no regression was computed due to the limited sample size, with combined DGT-plant data being available for soils AS and MZ only (Table S10, Table S12). The data for lettuce, wheat, and radish grown on these soils demonstrated that *δ*(^207^Pb/^206^Pb), *δ*(^208^Pb/^206^Pb), and *δ*(^206^Pb/^204^Pb) in plant tissues agreed with those assessed by DGT with respect to the uncertainty (Fig. 4e–p). For the uncontaminated soils TU, SL, and ST, comparison of Pb isotope ratios in plants with EDTA-extractable and total fractions showed substantial offsets between −15.6 and 33.9‰ (Table S10, Table S12). Moreover, the Pb isotopic composition of these soils was characterized by high uncertainty due to large variability between the sample replicates, resulting in little isotopic differentiation between soils and especially plants grown on these soils (Table S10, Table S12). This indicates that Pb isotopes may have limited power for precise source tracing in uncontaminated soil-plant systems, where minor differences in soil homogeneity and plant uptake may cause substantial isotopic variability. In comparison, in the contaminated soils AS and MZ, the isotopic differences between DGT-labile soil fractions and plants were low, with average *∆*(^207^Pb/^206^Pb)_DGT-plant_, *∆*(^208^Pb/^206^Pb)_DGT-plant_, and *∆*(^206^Pb/^204^Pb)_DGT-plant_ values of 0.20 ± 0.19‰, 0.26 ± 0.16‰, and −0.31 ± 0.57‰ (mean ± 2*s*, *n* = 8) for TK100 DGT, and −0.11 ± 0.30‰, −0.11 ± 0.48‰, and 0.15 ± 0.36‰ (mean ± 2*s*, *n* = 8) for Chelex DGT, respectively (Fig. 5b, Table S14). Thus, the close agreement was independent of the DGT technique used, as evidenced by the low average difference between the TK100 and Chelex DGT of 0.07 ± 0.82‰ (expressed as *∆*_TK100 DGT_−*∆*_Chelex DGT_; mean ± 2*s*, *n* = 24) across all assessed Pb isotope ratios. This demonstrates that plants accessed the same isotopically distinct labile Pb pools as those sampled by the DGT techniques and indicates that MDF during Pb translocation from soils AS and MZ to plants was not significant with respect to the uncertainty. Even within radish, large differences in Pb mass fractions between shoots and bulbs (Table S7) were not accompanied by significant isotopic differences (Fig. 5b, Table S12, Table S14), confirming the absence of relevant within-plant MDF under contaminated soil conditions.

From an applied perspective, these findings have direct relevance for applications such as food authentication, provenance tracing, and forensic case work, where bioavailable Sr and Pb isotope baselines are often based on the direct analysis of plant material [2, 25]. However, plants take up Sr and Pb from multiple and variably accessed soil layers, influenced by species-specific uptake or translocation processes, all of which can introduce variability and reduce isotopic discrimination power [14, 25, 26]. DGT, in contrast, provides a plant-independent assessment of the labile, bioavailable isotopic pool in soils, reducing biological and environmental variability while offering reproducible, low-uncertainty isotope ratios across soil types. This enables more standardized and representative assessments, supporting the establishment of robust bioavailable isotope baselines with improved comparability among sampling sites.

## Conclusions

Radiogenic isotope signatures of bioavailable Sr and Pb in soils are powerful tracers of biogeochemical processes, contamination sources, and material provenance. Conventional extraction methods can access these signatures but are resource-intensive, lack mechanistic specificity, and yield complex solutions that complicate isotopic analysis. This study demonstrates that DGT provides an effective alternative. The isotope signatures of Sr and Pb in soils assessed by DGT were geochemically site-specific and closely mirrored those in plants, confirming that the DGT-labile pool reflects the isotopic composition available for plant uptake for both elements. Concomitantly, contrasting elemental mass uptake patterns revealed distinct mechanisms: Sr uptake showed no correlation with the DGT-labile fraction, indicating mass flow control, whereas Pb uptake followed DGT-labile concentrations, consistent with diffusion control. Together, these findings show that combining DGT with both elemental and isotopic analyses provides a mechanistic framework for a more comprehensive appreciation of metal bioavailability in soils.

Besides mechanistic insights, DGT provides several analytical and practical advantages. First, effective matrix reduction via selective sampling by DGT minimizes purification needs and analytical interferences. For Pb, DGT eluates were sufficiently clean for direct isotopic analysis, while Sr still required column purification but with substantially lower matrix loads than conventional extracts. Among the tested methods, the TK100 DGT proved most effective, enabling quantitative assessment of both isotopic systems from the same sample. Moreover, DGT offers strong potential for field applications: it is portable, low-waste, and minimally invasive, requiring only ~ 2 g of soil per sampler. It can be deployed in undisturbed field soils, preserving in situ conditions, and is well suited for spatially resolved isotope ratio analysis in heterogeneous or remote environments. These assets position DGT as a powerful tool for establishing bioavailable isotopic baselines and resolving mixed geogenic and anthropogenic sources often masked in bulk soil analyses. Future studies should extend its application across a wider range of natural soil-plant systems under field conditions to fully assess their potential for isotope tracing in environmental forensics, food authentication, and archaeological provenance research.

## Supplementary Information

Below is the link to the electronic supplementary material.

Supplementary file1 Additional details on laboratory procedures, solutions, and materials; total soil and extract digestion; plant sample preparation and digestion; matrix separation; optimization procedures of MC-ICP-MS measurements; characteristics of soils; background equivalent concentrations, limits of detection, and limits of quantification of TK100 and Chelex DGT; elution parameters of automated matrix separation; instrumental parameters of MC-ICP-MS measurements; elemental and isotope ratio analysis results of certified reference materials; Sr and Pb mass fractions in plant tissues; relationships between Sr plant uptake and soil bioavailability indices; Ca/Sr, Rb/Sr, and Ca/Pb mass fraction ratios in DGT eluates and extracts; relative and absolute Sr and Pb isotope ratios in DGT-labile, extractable, and total soil fractions; relative and absolute Sr and Pb isotope ratios in lettuce, wheat, and radish tissues; isotopic differences of Sr and Pb between DGT-labile soil fractions and plant tissues. (PDF 472 KB)

## Data Availability

The datasets generated during the current study are available from the corresponding author on reasonable request.
