# Supplementary material for: Prediction of radiogenic Sr and Pb isotope signatures in plants using diffusive gradients in thin films
Source: Anal Bioanal Chem. 2026 Feb 2;418(5):1541–55. doi: 10.1007/s00216-026-06315-6 (PMC12909409; doi:10.1007/s00216-026-06315-6)
Supplement: Supplementary file 1 — Supplementary file1 Additional details on laboratory procedures, solutions, and materials; total soil and extract digestion; plant sample preparation and digestion; matrix separation; optimization procedures of MC-ICP-MS measurements; characteristics of soils; background equivalent concentrations, limits of detection, and limits of quantification of TK100 and Chelex DGT; elution parameters of automated matrix separation; instrumental parameters of MC-ICP-MS measurements; elemental and isotope ratio analysis results of certified reference materials; Sr and Pb mass fractions in plant tissues; relationships between Sr plant uptake and soil bioavailability indices; Ca/Sr, Rb/Sr, and Ca/Pb mass fraction ratios in DGT eluates and extracts; relative and absolute Sr and Pb isotope ratios in DGT-labile, extractable, and total soil fractions; relative and absolute Sr and Pb isotope ratios in lettuce, wheat, and radish tissues; isotopic differences of Sr and Pb between DGT-labile soil fractions and plant tissues. (PDF 472 KB) [file 216_2026_6315_MOESM1_ESM.pdf]

Supplementary Information

Prediction of Radiogenic Sr and Pb Isotope Signatures in Plants using Diffusive Gradients in Thin Films

Stefan Wagner[a,*], Jakob Santner[b], Markus Puschenreiter[c], Johanna Irrgeher[a], Thomas Prohaska[a]

[a] Montanuniversität Leoben, Department General, Analytical and Physical Chemistry, Chair of General and Analytical Chemistry, Franz-Josef-Straße 18, 8700, Leoben, Austria

[b] Justus Liebig University Giessen, Institute of Plant Nutrition, Heinrich-Buff-Ring 26-32, 35392, Giessen, Germany

[c] BOKU University, Department of Forest and Soil Sciences, Institute of Soil Research, Konrad-Lorenz-Straße 24, 3430, Tulln, Austria

[*]Corresponding author: stefan.wagner@unileoben.ac.at

**Contents**	**Page**
Text S1. Laboratory procedures, solutions, and materials	S-3
Text S2. Total soil and extract digestion procedures	S-4
Text S3. Plant sample preparation and digestion	S-5
Text S4. Matrix separation	S-6
Text S5. Daily optimization of MC-ICP-MS measurements	S-7
Table S1. Characteristics of soils	S-8
Table S2. Background equivalent concentrations (BECs), limits of detection (x_L), and limits of quantification (x_Q) of the TK100 DGT and Chelex DGT for Sr and Pb	S-9
Table S3. Elution parameters of the automated matrix separation	S-10
Table S4. Instrumental parameters of MC-ICP-MS measurements	S-11
Table S5. Elemental analysis results for CRMs	S-12
Table S6. Isotope ratio analysis results for CRMs	S-13
Table S7. Sr and Pb mass fractions (mg kg^{-1} dry weight) in lettuce, wheat, and radish tissues grown in soils	S-14
Table S8. Relationship between Sr plant uptake and soil bioavailability indices (TK100 DGT, NH_4NO_3, NH_4OAc) of the five experimental soils	S-15
Table S9. Mass fraction (*w*) ratios of Ca/Sr, Rb/Sr × 1000, and Ca/Pb in DGT eluates and soil extracts	S-16
Table S10. Relative Sr and Pb isotope ratios, expressed as δ-values, in DGT-labile, extractable, and total soil fractions	S-17
Table S11. Absolute Sr and Pb isotope ratios in DGT-labile, extractable, and total soil fractions	S-18
Table S12. Relative Sr and Pb isotope ratios, expressed as δ-values, in lettuce, wheat, and radish tissues grown in soils	S-19
Table S13. Absolute Sr and Pb isotope ratios in lettuce, wheat, and radish tissues grown in soils	S-20
Table S14. Isotopic differences, expressed as Δ-values, between DGT-labile (TK100 or Chelex) soil fractions and plant tissues	S-21
References	S-22

Text S1. Laboratory procedures, solutions, and materials

Soil and plant experiments, as well as matrix separation of Sr and Pb were performed at BOKU University (Institute of Soil Research, UFT Campus Tulln an der Donau), Austria. Mass spectrometric measurements were performed in cleanrooms (ISO class 8 according to ISO 14644-1) at both BOKU University and Montanuniversität Leoben (Chair of General and Analytical Chemistry), Austria.

Ultrapure water (σ = 0.055 µS cm^{-1}; MilliQ IQ 7000, Merck-Millipore, Darmstadt, Germany) was used in all experimental work. Ultrapure water and analytical reagent-grade HNO_3 (w = 65 %, Merck, Darmstadt, DE) used for sample preparation were additionally cleaned by single and double subboiling, respectively, in separate perfluoroalkoxy-polymer (PFA) distillation systems (DST-1000, Savillex, Eden Prairie, MN, USA). Trace metals basis HCl (w = 37 %, PanReac AppliChem, DE) was subboiled in a quartz distillation system (AHF Analysentechnik, Tübingen, DE). Polyethene (PE) and glass materials, as well as piston-type acrylonitrile butadiene styrene (ABS) DGT moldings (R-SDU; DGT Research Ltd., Lancaster, UK) were cleaned in two steps using HNO_3 (w = 10 % and w = 1 %, respectively). PFA vials used for matrix evaporation were cleaned using hot HNO_3 (w = 65 %) vapor in a PTFE cleaning system (Analab, Elemental Scientific, Omaha, USA). Following acid cleaning, all materials were rinsed with ultrapure water and dried in a metal-free class 100 laminar flow cabinet prior to use. If not stated otherwise, handling of DGT components and samples was carried out following clean trace analysis procedures as reported previously [1].

Subboiled HNO_3 and HCl, as well as H_2O_2 (w = 30 %, suprapur, Merck), HF (w = 40 %, for analysis, Merck), and H_3BO_3 (w = 4 %, suprapur, Merck) were used for digestion. Soil extraction solutions were prepared by dissolving ammonium nitrate (NH_4NO_3; ≥99 %, Sigma-Aldrich, DE), ammonium acetate (NH_4OAC; IUPAC name: ammonium ethanoate, $NH_4CH_3CO_2$; for analysis, ≥98.0 %, Merck), and ethylenediaminetetraacetic acid (EDTA; $C_{10}H_{14}N_2Na_2O_8 \times 2H_2O$; 99.0-101 %, Merck) in ultrapure water.

Single-element standards (c = 1 g L^{-1}) of Ca, Rb, Sr, In, and Pb were obtained from Inorganic Ventures (Christiansburg, USA). Multi-element standards for external calibration in ICP-MS measurements were prepared gravimetrically from a multi-element stock solution (ICP multielement standard Merck VI, Merck). For instrumental isotopic fractionation (IIF) correction, certified reference materials (CRMs) for Sr (SRM 987, high purity $SrCO_3$) and Pb (SRM 981, high purity Pb wire) with certified isotopic abundance ratios of $^{87}Sr/^{86}Sr$ = 0.71034 ± 0.00026 and $^{207}Pb/^{206}Pb$ = 0.91464 ± 0.00033, $^{208}Pb/^{206}Pb$ = 2.1681 ± 0.0008, and $^{206}Pb/^{204}Pb$ = 16.937 ± 0.011 (U, k = 2), respectively, were obtained from the National Institute of Standards and Technology (NIST, Gaithersburg, USA) [2, 3]. Preparation and handling of the unbranched DGA resin (particle size 50-100 µm; TrisKem International, Bruz, FR) used for matrix separation was performed as detailed in Retzmann et al. [4], except for soaking the resin in diluted ethanol (w = 30 %, absolute, Merck) instead of HNO_3 (w = 2 %) before column packing.

Text S2. Total soil and extract digestion procedures

Total soil digestion was performed using a method adapted from Prohaska et al. [5]. Briefly, ground soil samples (m = ~100 mg) were digested in a mixture of 5 mL HNO_3 (w = 65 %), 2 mL HCl (w = 37 %), and 1 mL HF (w = 40 %) for 30 min at 200 °C and 5.5×10^6 Pa in a microwave system (MARS 6, CEM, Charlotte, NC, USA). Subsequently, residual HF was neutralized by addition of 6 mL H_3BO_3, followed by 30 min at 170 °C and 5.5×10^6 Pa in the microwave. The total soil digests were diluted to obtain a total volume of ~50 mL using ultrapure water. For digestion of soil extracts, NH_4OAc and EDTA extract solutions (m = ~5 g) were weighed into 75 mL PTFE vessels and digested in a mixture of 5 mL HNO_3 (w = 65 %) and 1 mL H_2O_2 (w = 30 %) for 20 min at 200 °C and 5.5×10^6 Pa using the same microwave system as for total soil digestion. The extract digests were diluted to obtain a total volume of ~30 mL using ultrapure water.

Text S3. Plant sample preparation and digestion

Upon harvest, plant samples were rinsed with ultrapure water, blotted dry, and freeze-dried for seven days at -88 °C and 0.63 mbar (Alpha 2-4 LSCbasic, Christ, DE). Dried plant tissues were ground and homogenized using an analytical mill (A 11 basic, IKA, DE). Subsamples (m = ~150 mg) of the ground plant tissues were digested in a mixture of 3 mL HNO_3 (w = 65 %) and 0.76 mL H_2O_2 (w = 30 %) using the same microwave settings as for soil extract digestion (**Text S2**). Prior to the addition of H_2O_2, the samples were left overnight in HNO_3 to allow for extended reaction time. Digests were diluted to ~20 mL with ultrapure water and passed through a 0.45 µm pore-size PES syringe filter (Sartorius, DE) to remove non-dissolved silicates.

Text S4. Matrix separation

To obtain purified Sr and Pb fractions for isotope ratio analysis, soil extracts, total soil digests, and plant digests were processed by ion chromatography using an simultaneous Sr and Pb matrix separation procedure [6]. The method is based on an automated chromatographic system (prepFAST MC, Elemental Scientific, Omaha, USA) equipped with a self-packed 3 mL bed-volume column containing DGA resin (TrisKem International, Bruz, FR) with high affinity for Sr and Pb in HNO_3 (c = 2 mol L^{-1}). The method allows for simultaneous separation of Sr and Pb from Ca-rich matrices with quantitative analyte recovery and separate collection of the purified Sr and Pb fractions. Details of the prepFAST MC system and column packing are provided in Retzmann et al. [4] and Zimmermann et al. [6]. Separation performance of newly packed columns was verified in calibration mode of the prepFAST MC loading 3 mL of an in-house reference solution containing Sr (c = 0.25 mg L^{-1}), Pb (c = 0.05 mg L^{-1}), Ca (c = 100 mg L^{-1}), and Rb (c = 1 mg L^{-1}). Prior to sample loading, the matrix of sample solutions was adjusted to a constant HNO_3 concentration (c = 2 mol L^{-1}) using either HNO_3 (w = 65%) or ultrapure water. All NH_4NO_3 extracts were diluted by a factor of 20 to reduce the on-column salt load for interference-free ion extraction. The NH_4OAc and EDTA soil extract digests, total soil digests, and plant digests were diluted by varying factors aiming at Sr and Pb levels in separated eluate fractions >20 µg L^{-1} and >2 µg L^{-1}, respectively, at a total sample load volume between 0.5-10 mL. To avoid any Sr and Pb overlap during elution, the total on-column Ca load was kept <500 µg [6]. If Sr or Pb concentrations in the respective eluate fractions were <20 µg L^{-1} or <2 µg L^{-1}, respectively, matrix evaporation was performed in PFA vials at 90 °C on a hotplate. The eluates were evaporated until a remaining volume of approximately 50 µL and then redissolved in HNO_3 (c = 2 %). Elution parameters are shown in **Table S3**.

Text S5. Daily optimization of MC-ICP-MS measurements

The preamplifier gains on Faraday cups were calibrated daily and the detector baselines were accounted for via repeated on-peak zero measurements in each analytical batch. Before each measurement sequence, the operating conditions were optimized for maximum analyte sensitivity, signal stability, and peak shape using solutions of SRM 987 for Sr and SRM 981 for Pb at the target sample concentrations. Typical optimizations included adjustment of torch position, desolvator and nebulizer Ar gas flows, ion lens voltages, and zoom optics to achieve stable beam intensities and consistent isotope ratios across all analytical runs.

Table S1. Characteristics of soils [a]

Parameter	Unit	TU	SL	ST	AS	MZ	PR
Site	-	Tulln	Siebenlinden	Stetting	Arnoldstein	Mežica	Příbram
Country	-	AT	AT	DE	AT	SI	CZ
Latitude	-	48.318889° N	48.675222° N	48.635778° N	46.553639° N	46.545944° N	49.719417° N
Longitude	-	16.069333° E	14.998889° E	13.264167° E	13.690000° E	14.937472° E	14.013111° E
Soil type [b]	-	Calcic chernozem	Acidic cambisol	Acidic cambisol	Acidic cambisol	Calcic cambisol	Acidic fluvisol
pH_{CaCl2}	-	7.3 ± <0.1	4.5 ± <0.1	4.8 ± <0.1	4.8 ± <0.1	7.0 ± <0.1	5.0 ± <0.1
$w(CaCO_3)$	g kg^{-1}	83 ± 3	13 ± 5	17 ± 6	$<x_L$	115 ± 26	$<x_L$
$w(C)_{organic}$	g kg^{-1}	26.8 ± 0.4	20.0 ± 1.6	9.85 ± 0.16	25.3 ± 0.1	67.1 ± 0.4	21.5 ± 0.1
Sand	g kg^{-1}	295 ± 7	532 ± 8	331 ± 6	409 ± 2	486 ± 17	598 ± 11
Silt	g kg^{-1}	438 ± 3	333 ± 8	421 ± 1	466 ± 8	432 ± 17	320 ± 11
Clay	g kg^{-1}	268 ± 4	136 ± <1	248 ± 5	126 ± 7	82 ± <1	82 ± <1
CEC	mmol$_c$ kg^{-1}	340 ± 11	65 ± 2	91 ± 1	67 ± 7	247 ± 6	51 ± 1
WHC	g kg^{-1}	454 ± 16	447 ± 1	467 ± 10	446 ± 12	460 ± 3	418 ± 2
$w(Sr)_{total}$	mg kg^{-1}	130 ± 2	163 ± 10	79.2 ± 0.4	87.5 ± 2.0	149 ± 3	52.0 ± 0.7
$w(Pb)_{total}$	mg kg^{-1}	28.0 ± 2.8	45.9 ± 1.4	27.6 ± 0.2	1049 ± 19	2232 ± 45	5009 ± 113
$w(Sr)_{NH4NO3}$	mg kg^{-1}	7.83 ± 0.46	2.45 ± 0.06	2.14 ± 0.03	2.42 ± 0.11	5.00 ± 0.12	2.20 ± 0.03
$w(Sr)_{NH4OAc}$	mg kg^{-1}	8.85 ± 0.07	2.66 ± 0.05	2.47 ± 0.03	2.52 ± 0.05	5.38 ± 0.05	1.87 ± 0.03
$w(Pb)_{NH4NO3}$	mg kg^{-1}	$<x_L$	0.06 ± <0.01	0.02 ± <0.01	10.4 ± 0.4	0.50 ± 0.01	12.8 ± 0.3
$w(Pb)_{EDTA}$	mg kg^{-1}	10.4 ± 0.6	6.25 ± 0.19	4.04 ± 0.21	693 ± 17	1419 ± 28	2810 ± 73
$w(Rb)_{total}$/ $w(Sr)_{total}$	-	0.79 ± 0.01	1.06 ± 0.04	2.20 ± 0.02	1.18 ± 0.01	0.57 ± 0.02	0.90 ± 0.01
Lithological background	-	Clastic sediment	Muscovite-biotite granite	Paragneiss, migmatite	Glacial sediment	Undifferentiated sediment	Shale, mudstone
Geological age	-	Quaternary	Carboniferous	Neoproterozoic	Paleozoic	Neoproterozoic	Paleozoic

[a] Values are means ± *s* with *n* = 3, except for clay content and WHC where *n* = 2 (physical replicates). [b] According to World Reference Base for Soil Resources (WRB) [7].

Table S2. Background equivalent concentrations (BECs), limits of detection (x_L), and limits of quantification (x_Q) of the TK100 DGT and Chelex DGT for Sr and Pb

**DGT**	**Sr**						**Pb**					
	**Disc loading / (ng disc^{-1})**			**c_{DGT} / (µg L^{-1})**			**Disc loading / (ng disc^{-1})**			**c_{DGT} / (µg L^{-1})**		
	BEC	x_L	x_Q	BEC	x_L	x_Q	BEC	x_L	x_Q	BEC	x_L	x_Q
TK100	4.37	3.59	12.0	0.25	0.20	0.68	13.6	10.8	35.9	0.59	0.47	1.57
Chelex	3.76	1.18	3.92	0.21	0.07	0.22	1.09	1.49	4.95	0.05	0.06	0.22

Table S3. Elution parameters of the automated matrix separation using the prepFAST MC

Step	Volume / mL	Flow rate / (mL min^{-1})	Reagent	Reagent concentration / (mol L^{-1})
Condition column	10	2	HNO_3	2
Load sample	0.5-10	1	HNO_3	2
Elute matrix	8	2	HNO_3	2
Elute Sr	7	1	HNO_3	0.2
Elute Pb	5-7	1	HNO_3	5
Elute Ca/wash column	15	2	HCl	0.1

Table S4. Instrumental parameters of MC-ICP-MS measurements

Parameter	Sr	Pb
	Nu Sapphire (SP017, Nu Instruments) [a]	
RF power / W	1300	
Coolant flow / L min^{-1}	13	
Auxiliary flow / L min^{-1}	0.9	
Nebulizer pressure / psi	29-32	
Interface cones	Ni, dry	
Resolution mode ($m/\Delta m$)	Low (~300)	
Sample introduction system	Apex Omega (ESI) or Aridus 3 (Teledyne CETAC) with PFA nebulizer (ESI)	
Scan mode	Static batch analysis	
Integration time / s	10	
Number of cycles per block	10	
Number of blocks	6	
Blank acquisition mode	Measure zero (on-peak)	
Blank integration time / s	300	
Cup configuration (cup: m/z)	L5: 82, L4: 83, L2: 84, Ax: 85, H2: 86, H4: 87, H6: 88, H7: 89, H8: 90, H9: 91, H10: 92	L3: 202, L2: 203, L1: 204, Ax: 205, H1: 206, H2: 207, H3: 208
Preamplifier feedback resistor	10^{11} Ω	10^{11} Ω
Average sensitivity / V/(µg g^{-1})	900	1500
**Parameter**	**Nu Plasma HR (NP048, Nu Instruments) [b]**	
RF power / W	1300	
Coolant flow / L min^{-1}	13	
Auxiliary flow / L min^{-1}	0.9	
Nebulizer pressure / psi	26-31	
Interface cones	Ni, dry	
Resolution mode ($m/\Delta m$)	Low (~300)	
Sample introduction system	Aridus II (Teledyne CETAC) with PFA nebulizer (ESI)	
Scan mode	Static batch analysis	
Integration time / s	10	
Number of cycles per block	10	
Number of blocks	6	
Blank acquisition mode	Measure zero (on-peak)	
Blank integration time / s	300	
Cup configuration (cup: m/z)	L5: 83, L4: 84, L3: 85, L2: 86, Ax: 87, H2: 88, H4: 89, H5: 90, H6: 91	L2: 202, L1: 203, Ax: 204, H1: 205, H2: 206, H3: 207, H4: 208
Preamplifier feedback resistor	10^{11} Ω	10^{11} Ω
Average sensitivity / V/(µg g^{-1})	160	500

[a] Used for the following isotope ratio measurements: Sr: TK100 DGT eluates; Pb: TK100 DGT eluates, wheat digests (soils TU, SL, ST), radish shoot digests, radish bulb digests (soils TU, SL, ST).

[b] Used for the following isotope ratio measurements: Sr: NH_4NO_3 extracts, NH_4OAc extracts, total digests, lettuce digests, wheat digests, radish shoot digests, radish bulb digests; Pb: Chelex DGT eluates, NH_4NO_3 extracts, EDTA extracts, total soil digests, lettuce digests, wheat digests (soils AS and MZ), radish bulb digests (soils AS and MZ).

Table S5. Elemental analysis results for certified reference materials (CRMs) [a]

CRM	Matrix	Sr			Pb		
		Experimental $w(Sr)$ / (mg kg^{-1})	Certified $w(Sr)$ / (mg kg^{-1})	Recovery / %	Experimental $w(Pb)$ / (mg kg^{-1})	Certified $w(Pb)$ / (mg kg^{-1})	Recovery / %
ISE 885	Soil	72.3 ± 2.2	66.3 ± 7.8	109 ± 3	20.7 ± 0.8	20.2 ± 4.6	102 ± 4
SRM 1547	Peach leaves	62.7 ± 5.8	53.0 ± 5.0	118 ± 11	0.84 ± 0.09	0.869 ± 0.018	97.1 ± 9.8
IPE 200	Maize shoots	14.5 ± 1.9	13.5 ± 1.6	107 ± 14	0.30 ± 0.07	0.292 ± 0.155	104 ± 25

[a] Experimental values are means ± 2*s* (*n* = 3 for ISE 885; *n* = 15 for SRM 1547; *n* = 12 for IPE 200). Certified values were taken from the CRM certificates [8-10], with errors showing 2*s* for ISE 885 and IPE 200 and expanded uncertainties (*U*, *k* = 2) for SRM 1547.

Table S6. Isotope ratio analysis results for certified reference materials (CRMs) and quality controls (QCs) [a]

Isotope ratio	Unit	SRM 987 (n = 60)	SRM 981 (n = 59)	QC-SRM 987 (n = 52)	QC-SRM 981 (n = 57)	ISE 885 (n = 3)	SRM 1547 (n = 15)	IPE 200 (n = 14)
$\delta(^{87}Sr/^{86}Sr)$	‰	0.00 ± 0.04	n.a.	0.06 ± 0.22	n.a.	11.6 ± 0.2	4.55 ± 0.04	-0.90 ± 0.08
$n(^{87}Sr)/n(^{86}Sr)$	-	0.71035 ± 0.00008	n.a.	0.71043 ± 0.00020	n.a.	0.71855 ± 0.00018	0.71356 ± 0.00005	0.70967 ± 0.00008
$\delta(^{207}Pb/^{206}Pb)$	‰	n.a.	0.00 ± 0.21	n.a.	-0.19 ± 0.41	-87.6 ± 0.8	-98.5 ± 0.6	-63.8 ± 3.6
$n(^{207}Pb)/n(^{206}Pb)$	-	n.a.	0.91464 ± 0.00020	n.a.	0.91447 ± 0.00037	0.83449 ± 0.00072	0.82456 ± 0.00054	0.85629 ± 0.00325
$\delta(^{208}Pb/^{206}Pb)$	‰	n.a.	-0.01 ± 0.46	n.a.	-0.15 ± 0.59	-46.5 ± 1.0	-55.3 ± 0.5	-34.8 ± 3.0
$n(^{208}Pb)/n(^{206}Pb)$	-	n.a.	2.16808 ± 0.00100	n.a.	2.16778 ± 0.00128	2.06731 ± 0.00219	2.04812 ± 0.00100	2.09275 ± 0.00648
$\delta(^{206}Pb/^{204}Pb)$	‰	n.a.	0.00 ± 0.45	n.a.	0.14 ± 1.05	108 ± 1	121 ± 3	77.0 ± 4.7
$n(^{206}Pb)/n(^{204}Pb)$	-	n.a.	16.93635 ± 0.01158	n.a.	16.93850 ± 0.01996	18.76860 ± 0.00642	19.00246 ± 0.04027	18.24420 ± 0.08172

[a] Values are means ± 2s. n.a. = not analyzed.

Table S7. Sr and Pb mass fractions (w, in mg kg^{-1} dry weight) in lettuce, wheat, and radish tissues grown in soils [a]

Soil	Lettuce (shoot)	Wheat (shoot)	Radish (shoot)	Radish (bulb)	*p*	Lettuce (shoot)	Wheat (shoot)	Radish (shoot)	Radish (bulb)	*p*
	w(Sr) / (mg kg^{-1})					w(Pb) / (mg kg^{-1})				
TU	25.2 ± 2.8	9.69 ± 1.03	88.6 ± 11.6	20.9 ± 2.9	***	0.09 ± 0.04	0.05 ± 0.01	0.10 ± 0.03	0.19 ± 0.16	ns
	a,w	a,x	y	a,w		a	a	a		
SL	37.2 ± 3.8	24.0 ± 1.6	74.6 ± 15.2	28.1 ± 5.4	**	0.57 ± 0.20	0.13 ± 0.04	0.12 ± 0.01	0.20 ± 0.14	*
	b,w	b,x	y	a,wx		a	a	a		
ST	21.4 ± 2.5	7.11 ± 0.56	70.9 ± 8.4	12.7 ± 1.8	***	0.10 ± 0.03	0.06 ± 0.01	0.10 ± 0.03	0.09 ± 0.03	ns
	a,w	c,x	y	b,z		a	a	a		
AS	27.8 ± 2.5	32.6 ± 4.3	90.1 ± 23.5	40.3 ± 16.3	**	38.9 ± 17.0	26.2 ± 4.8	26.6 ± 14.0	434 ± 272	ns
	a,w	bd,w	x	ab,wx		ab	b	ab		
MZ	37.4 ± 2.2	30.0 ± 2.0	86.7 ± 5.0	24.1 ± 11.1	***	8.37 ± 2.71	5.64 ± 2.67	10.2 ± 3.4	37.0 ± 17.4	ns
	b,w	d,x	y	ab,wx		b	a	b		
p	***	***	ns	**		**	***	**	**	

[a] Values are means ± s (n = 4). Asterisks denote significance: * = $p < 0.05$, ** = $p < 0.01$, *** = $p < 0.001$. ns = not significant ($p > 0.05$). a, b, c, d = different letters along columns denote significant differences ($p < 0.05$) between soils within the same plant group; w, x, y, z = different letters along rows denote significant differences ($p < 0.05$) between plants within the same soil group.

Table S8. Relationships between Sr plant uptake and soil bioavailability indices (TK100 DGT, NH_4NO_3, NH_4OAc) of the five experimental soils [a]

Index		Lettuce (shoot)	Wheat (shoot)	Radish (shoot)	Radish (bulb)
TK100 DGT	*r*	0.296	-0.082	0.346	-0.288
	p	0.284	0.771	0.207	0.298
NH_4NO_3	*r*	0.033	-0.297	0.350	-0.225
	p	0.907	0.283	0.201	0.421
NH_4OAc	*r*	-0.007	-0.339	0.331	-0.260
	p	0.980	0.216	0.229	0.349

[a] *r* = correlation coefficient; *p* = level of significance (Pearson correlation)

Table S9. Mass fraction (w) ratios of Ca/Sr, Rb/Sr × 1000, and Ca/Pb in DGT eluates and soil extracts [a]

Soil	$TK100_{DGT}$	NH_4NO_3	NH_4OAc	*p*	$TK100_{DGT}$	NH_4NO_3	NH_4OAc	*p*	$TK100_{DGT}$	$Chelex_{DGT}$	NH_4NO_3	EDTA	*p*
	w(Ca)/w(Sr)				w(Rb)/w(Sr) × 1000				w(Ca)/w(Pb)				
TU	71.9 ± 23.1	361 ± 9	367 ± 3	***	0.06 ± 0.04	20.0 ± <0.1	17.1 ± 0.2	***	n.a.	n.a.	n.a.	2255 ± 185	n.a.
SL	49.3 ± 6.3	245 ± 3	224 ± 1	***	4.91 ± 0.57	509 ± 4	516 ± 2	***	n.a.	n.a.	n.a.	147 ± 6	n.a.
ST	64.7 ± 7.3	360 ± 1	318 ± 3	***	9.69 ± 1.57	735 ± 1	681 ± 2	***	n.a.	n.a.	n.a.	287 ± 10	n.a.
AS	54.1 ± 6.3	246 ± <1	233 ± 2	***	7.03 ± 1.05	308 ± 1	314 ± 1	***	12.7 ± 13.8	27.4 ± 1.0	57.7 ± 0.7	1.29 ± 0.04	***
MZ	51.1 ± 12.7	450 ± 2	494 ± 6	***	0.33 ± 0.09	84.0 ± 0.6	82.9 ± 1.0	***	176 ± 196	217 ± 44	4532 ± 12	14.5 ± 0.3	***
PR	35.7 ± 5.2	259 ± 1	250 ± 1	***	2.70 ± 0.59	168 ± 1	188 ± 1	***	3.79 ± 4.19	36.3 ± 2.5	44.5 ± 0.4	0.06 ± <0.01	***

[a] Values are means ± s (n = 3). Asterisks denote significance: *** = $p < 0.001$. n.a. = not analyzed.

Table S10. Relative Sr and Pb isotope ratios, expressed as δ-values, in DGT-labile, extractable, and total soil fractions [a]

Soil	$TK100_{DGT}$	NH_4NO_3	NH_4OAc	Total	$TK100_{DGT}$	$Chelex_{DGT}$	NH_4NO_3	EDTA	Total
	$\delta(^{87}Sr/^{86}Sr)$ / ‰				$\delta(^{207}Pb/^{206}Pb)$ / ‰				
TU	-1.29 ± 0.23	-1.12 ± 0.23	-1.07 ± 0.26	9.24 ± 0.42	n.a.	n.a.	n.a.	-79.6 ± 3.4	-85.0 ± 1.8
	a,w	a,w	a,w	a,x				a,w	a,x
SL	12.7 ± 0.3	12.9 ± 0.2	12.9 ± 0.3	10.6 ± 0.5	n.a.	n.a.	n.a.	-73.2 ± 1.2	-76.9 ± 2.1
	b,w	b,w	b,w	b,x				b,w	b,x
ST	2.66 ± 0.23	2.73 ± 0.23	2.87 ± 0.27	18.6 ± 0.4	n.a.	n.a.	n.a.	-81.4 ± 0.3	-92.0 ± 1.4
	c,w	c,w	c,w	c,x				a,w	c,x
AS	0.41 ± 0.23	0.44 ± 0.23	0.55 ± 0.26	19.6 ± 0.2	-67.0 ± 0.3	-67.2 ± 0.2	-67.2 ± 0.4	-67.1 ± 0.3	-67.4 ± 0.2
	d,w	d,w	d,w	d,x	a	a	a	c	d
MZ	-1.30 ± 0.22	-1.18 ± 0.23	-1.12 ± 0.27	5.31 ± 0.47	-67.3 ± 0.4	-67.7 ± 0.3	-67.4 ± 0.3	-67.4 ± 0.3	-67.6 ± 0.2
	a,w	a,w	a,w	e,x	a	b	a	c	d
PR	2.11 ± 0.35	2.04 ± 0.23	2.19 ± 0.29	12.7 ± 0.4	-58.2 ± 0.3	-58.3 ± 0.3	-58.2 ± 0.5	-58.1 ± 0.3	-58.3 ± 0.2
	e,w	e,w	e,w	f,x	b	c	b	d	e
	-				$\delta(^{208}Pb/^{206}Pb)$ / ‰				
TU	-	-	-	-	n.a.	n.a.	n.a.	-43.4 ± 2.1	-45.0 ± 1.5
								a	a
SL	-	-	-	-	n.a.	n.a.	n.a.	-37.8 ± 0.9	-29.3 ± 1.3
								b,w	b,x
ST	-	-	-	-	n.a.	n.a.	n.a.	-46.0 ± 0.3	-46.9 ± 1.6
								c	a
AS	-	-	-	-	-32.4 ± 0.3	-32.6 ± 0.2	-32.6 ± 0.6	-32.4 ± 0.3	-32.5 ± 0.2
					a	a	a	d	c
MZ	-	-	-	-	-32.4 ± 0.3	-32.9 ± 0.3	-32.5 ± 0.3	-32.4 ± 0.3	-32.5 ± 0.2
					a,w	a,x	a,wx	d,w	c,w
PR	-	-	-	-	-27.6 ± 0.4	-27.7 ± 0.3	-27.4 ± 0.8	-27.3 ± 0.3	-27.5 ± 0.3
					b	b	b	e	d
	-				$\delta(^{206}Pb/^{204}Pb)$ / ‰				
TU	-	-	-	-	n.a.	n.a.	n.a.	97.5 ± 4.5	105 ± 2
								a,w	a,x
SL	-	-	-	-	n.a.	n.a.	n.a.	88.4 ± 1.4	93.4 ± 2.5
								b,w	b,x
ST	-	-	-	-	n.a.	n.a.	n.a.	101 ± <1	115 ± 2
								a,w	c,x
AS	-	-	-	-	83.4 ± 0.3	83.7 ± 0.3	83.5 ± 0.5	83.8 ± 0.3	84.2 ± 0.3
					a,w	a,w	a,w	c,w	d,x
MZ	-	-	-	-	83.5 ± 0.8	84.0 ± 0.2	84.1 ± 0.3	84.4 ± 0.3	84.6 ± 0.3
					a,w	a,w	a,wx	d,xy	e,y
PR	-	-	-	-	68.4 ± 0.4	68.4 ± 0.3	68.6 ± 0.9	68.6 ± 0.3	68.5 ± 0.4
					b	b	b	e	f

[a] Values are means (n = 3) ± U (k = 2). TK100 DGT and NH_4NO_3 data for soils SL, AS, and MZ was re-evaluated from Wagner et al. [1] using isotopic fractionation correction via $^{88}Sr/^{86}Sr$. n.a. = not analyzed. a, b, c, d, e, f = different letters along columns denote significant differences between soils within the same soil assessment method group; w, x, y = different letters along rows denote significant differences between soil assessment methods within the same soil group. Statistical differences between two mean values were assessed considering their expanded uncertainties (U, k = 2) [11].

Table S11. Absolute Sr and Pb isotope ratios in DGT-labile, extractable, and total soil fractions [a]

Soil	$TK100_{DGT}$	NH_4NO_3	NH_4OAc	Total	$TK100_{DGT}$	$Chelex_{DGT}$	NH_4NO_3	EDTA	Total
	$n(^{87}Sr)/n(^{86}Sr)$				$n(^{207}Pb)/n(^{206}Pb)$				
TU	0.70963 ± 0.00022	0.70955 ± 0.00013	0.70953 ± 0.00016	0.71690 ± 0.00028	n.a.	n.a.	n.a.	0.84188 ± 0.00307	0.83689 ± 0.00163
SL	0.71958 ± 0.00038	0.71953 ± 0.00013	0.71960 ± 0.00016	0.71782 ± 0.00031	n.a.	n.a.	n.a.	0.84773 ± 0.00108	0.84429 ± 0.00190
ST	0.71192 ± 0.00086	0.71229 ± 0.00014	0.71241 ± 0.00017	0.72353 ± 0.00025	n.a.	n.a.	n.a.	0.84023 ± 0.00031	0.83048 ± 0.00128
AS	0.71086 ± 0.00026	0.71065 ± 0.00013	0.71073 ± 0.00016	0.72427 ± 0.00015	0.85334 ± 0.00033	0.85316 ± 0.00021	0.85316 ± 0.00052	0.85327 ± 0.00025	0.85300 ± 0.00041
MZ	0.70964 ± 0.00022	0.70950 ± 0.00013	0.70951 ± 0.00016	0.71408 ± 0.00032	0.85313 ± 0.00033	0.85273 ± 0.00029	0.85298 ± 0.00048	0.85295 ± 0.00025	0.85278 ± 0.00041
PR	0.71195 ± 0.00036	0.71177 ± 0.00013	0.71186 ± 0.00018	0.71930 ± 0.00029	0.86140 ± 0.00033	0.86129 ± 0.00025	0.86142 ± 0.00058	0.86150 ± 0.00025	0.86134 ± 0.00041
	-				$n(^{208}Pb)/n(^{206}Pb)$				
TU	-	-	-	-	n.a.	n.a.	n.a.	2.07397 ± 0.00446	2.07048 ± 0.00331
SL	-	-	-	-	n.a.	n.a.	n.a.	2.08622 ± 0.00182	2.10456 ± 0.00280
ST	-	-	-	-	n.a.	n.a.	n.a.	2.06838 ± 0.00046	2.06646 ± 0.00351
AS	-	-	-	-	2.09792 ± 0.00035	2.09748 ± 0.00025	2.09731 ± 0.00115	2.09785 ± 0.00037	2.09759 ± 0.00029
MZ	-	-	-	-	2.09793 ± 0.00064	2.09667 ± 0.00058	2.09765 ± 0.00053	2.09784 ± 0.00036	2.09757 ± 0.00021
PR	-	-	-	-	2.10832 ± 0.00043	2.10796 ± 0.00048	2.10872 ± 0.00170	2.10884 ± 0.00028	2.10837 ± 0.00042
	-				$n(^{206}Pb)/n(^{204}Pb)$				
TU	-	-	-	-	n.a.	n.a.	n.a.	18.58793 ± 0.07785	18.71407 ± 0.03173
SL	-	-	-	-	n.a.	n.a.	n.a.	18.43141 ± 0.03373	18.51719 ± 0.03337
ST	-	-	-	-	n.a.	n.a.	n.a.	18.64132 ± 0.00303	18.88559 ± 0.02039
AS	-	-	-	-	18.34875 ± 0.00850	18.35671 ± 0.00649	18.35426 ± 0.00300	18.35645 ± 0.00119	18.36612 ± 0.01126
MZ	-	-	-	-	18.35968 ± 0.00699	18.36292 ± 0.01178	18.36289 ± 0.00590	18.36650 ± 0.00150	18.37138 ± 0.00533
PR	-	-	-	-	18.09458 ± 0.00198	18.09463 ± 0.00036	18.09883 ± 0.01477	18.09996 ± 0.00123	18.09979 ± 0.00536

[a] Values are means (n = 3) ± U (k = 2). TK100 DGT and NH_4NO_3 data for soils SL, AS, and MZ was re-evaluated from Wagner et al. [1] using isotopic fractionation correction via $^{88}Sr/^{86}Sr$. n.a. = not analyzed.

Table S12. Relative Sr and Pb isotope ratios, expressed as δ-values, in lettuce, wheat, and radish tissues grown in soils [a]

Soil	Lettuce (shoot)	Wheat (shoot)	Radish (shoot)	Radish (bulb)	Lettuce (shoot)	Wheat (shoot)	Radish (shoot)	Radish (bulb)
	$\delta(^{87}Sr/^{86}Sr)$ / ‰				$\delta(^{207}Pb/^{206}Pb)$ / ‰			
TU	-1.12 ± 0.26	-1.08 ± 0.25	-1.12 ± 0.17	-1.05 ± 0.20	-60.8 ± 6.1	-59.2 ± 8.9	n.a.	-75.5 ± 13.3
	a	a	a	a	a,w	a,w		abc,x
SL	12.5 ± 0.4	13.0 ± 0.3	13.2 ± 0.2	13.4 ± 0.3	-71.3 ± 21.3	-66.4 ± 3.4	n.a.	-74.2 ± 2.1
	b,w	b,x	b,x	b,x	ab,wx	abc,x		a,y
ST	2.78 ± 0.31	2.98 ± 0.26	2.94 ± 0.18	3.23 ± 0.39	-68.0 ± 8.5	-67.9 ± 3.9	n.a.	-77.5 ± 9.8
	c	c	c	c	ab	abc		a
AS	0.54 ± 0.53	0.57 ± 0.25	0.57 ± 0.18	0.72 ± 0.34	-67.2 ± 0.4	-67.2 ± 0.2	-67.1 ± 0.4	-67.3 ± 0.2
	d	d	d	d	b	b		b
MZ	-1.01 ± 0.27	-0.96 ± 0.25	-1.07 ± 0.18	-0.94 ± 0.30	-67.5 ± 0.5	-67.5 ± 0.2	-67.3 ± 0.4	-67.6 ± 0.2
	a	a	a	a	b	c		c
	-				$\delta(^{208}Pb/^{206}Pb)$ / ‰			
TU	-	-	-	-	-31.6 ± 4.4	-31.3 ± 4.6	n.a.	-40.6 ± 7.3
					abc,w	a,w		a,x
SL	-	-	-	-	-32.8 ± 8.9	-34.1 ± 2.7	n.a.	-38.0 ± 1.2
					abc,wx	ab,w		a,x
ST	-	-	-	-	-35.6 ± 2.4	-36.3 ± 1.1	n.a.	-43.1 ± 5.3
					ab,w	b,w		a,x
AS	-	-	-	-	-32.6 ± 0.7	-32.7 ± 0.2	-32.7 ± 0.5	-32.8 ± 0.3
					c	a		b
MZ	-	-	-	-	-32.5 ± 1.0	-32.6 ± 0.3	-32.5 ± 0.5	-32.7 ± 0.2
					c	a		b
	-				$\delta(^{206}Pb/^{204}Pb)$ / ‰			
TU	-	-	-	-	72.6 ± 7.5	71.1 ± 11.7	n.a.	92.1 ± 17.8
					a,w	a,wx		ab,x
SL	-	-	-	-	86.0 ± 27.0	80.1 ± 4.4	n.a.	89.3 ± 3.2
					ab,wx	ab,w		a,x
ST	-	-	-	-	82.3 ± 10.1	82.16 ± 5.3	n.a.	94.6 ± 12.6
					ab	ab		ab
AS	-	-	-	-	83.5 ± 1.1	83.5 ± 0.6	83.3 ± 0.7	83.5 ± 0.6
					b	b		b
MZ	-	-	-	-	84.2 ± 1.4	84.0 ± 0.7	83.7 ± 0.8	84.1 ± 0.6
					b	b		b

[a] Values are means (n = 4) ± U (k = 2). n.a. = not analyzed. a, b, c, d = different letters along columns denote significant differences between soils within the same plant group; w, x, y = different letters along rows denote significant differences between plants within the same soil group. Statistical differences between two mean values were assessed considering their expanded uncertainties (U, k = 2) [11].

Table S13. Absolute Sr and Pb isotope ratios in lettuce, wheat, and radish tissues grown in soils [a]

Soil	Lettuce (shoot)	Wheat (shoot)	Radish (shoot)	Radish (bulb)	Lettuce (shoot)	Wheat (shoot)	Radish (shoot)	Radish (bulb)
	$n(^{87}Sr)/n(^{86}Sr)$				$n(^{207}Pb)/n(^{206}Pb)$			
TU	0.70955 ± 0.00016	0.70958 ± 0.00014	0.70958 ± 0.00009	0.70959 ± 0.00011	0.85907 ± 0.00557	0.86047 ± 0.00406	n.a.	0.84561 ± 0.01220
SL	0.71919 ± 0.00025	0.71962 ± 0.00016	0.71975 ± 0.00010	0.71983 ± 0.00021	0.84947 ± 0.01951	0.85396 ± 0.00309	n.a.	0.84676 ± 0.00196
ST	0.71233 ± 0.00019	0.71247 ± 0.00015	0.71244 ± 0.00010	0.71263 ± 0.00026	0.85245 ± 0.00775	0.85257 ± 0.00359	n.a.	0.84378 ± 0.00897
AS	0.71071 ± 0.00036	0.71073 ± 0.00014	0.71075 ± 0.00010	0.71086 ± 0.00021	0.85322 ± 0.00034	0.85317 ± 0.00015	0.85362 ± 0.00175	0.85307 ± 0.00014
MZ	0.70958 ± 0.00016	0.70964 ± 0.00014	0.70958 ± 0.00010	0.70967 ± 0.00018	0.85295 ± 0.00044	0.85291 ± 0.00016	0.85236 ± 0.00296	0.85281 ± 0.00014
	-				$n(^{208}Pb)/n(^{206}Pb)$			
TU	-	-	-	-	2.09951 ± 0.00950	2.10032 ± 0.00989	n.a.	2.08014 ± 0.01587
SL	-	-	-	-	2.09705 ± 0.01936	2.09427 ± 0.00589	n.a.	2.08579 ± 0.00262
ST	-	-	-	-	2.09093 ± 0.00512	2.08949 ± 0.00238	n.a.	2.07474 ± 0.01158
AS	-	-	-	-	2.09747 ± 0.00154	2.09728 ± 0.00050	2.09783 ± 0.00233	2.09709 ± 0.00054
MZ	-	-	-	-	2.09760 ± 0.00217	2.09734 ± 0.00060	2.09633 ± 0.00506	2.09726 ± 0.00050
	-				$n(^{206}Pb)/n(^{204}Pb)$			
TU	-	-	-	-	18.17979 ± 0.12681	18.15000 ± 0.18556	n.a.	18.50479 ± 0.28502
SL	-	-	-	-	18.39129 ± 0.47056	18.29869 ± 0.07738	n.a.	18.45277 ± 0.05457
ST	-	-	-	-	18.32777 ± 0.17258	18.33453 ± 0.08766	n.a.	18.54388 ± 0.22006
AS	-	-	-	-	18.35154 ± 0.01907	18.35113 ± 0.01036	18.33976 ± 0.03529	18.35068 ± 0.01089
MZ	-	-	-	-	18.36855 ± 0.02719	18.36228 ± 0.01854	18.37052 ± 0.06262	18.36029 ± 0.01139

[a] Values are means (n = 4) ± U (k = 2). n.a. = not analyzed.

Table S14. Isotopic differences, expressed as Δ-values, of Sr and Pb between TK100 and/or Chelex DGT-labile soil fractions and plant tissues [a]

Soil	TK100 DGT				Chelex DGT			
	**Lettuce (shoot)**	**Wheat (shoot)**	**Radish (shoot)**	**Radish (bulb)**	**Lettuce (shoot)**	**Wheat (shoot)**	**Radish (shoot)**	**Radish (bulb)**
	**$\Delta(^{87}Sr/^{86}Sr)_{DGT\text{-}plant}$ / ‰**				**$\Delta(^{87}Sr/^{86}Sr)_{DGT\text{-}plant}$ / ‰**			
TU	-0.17 ± 0.35	-0.21 ± 0.34	-0.17 ± 0.29	-0.24 ± 0.30	n.a.	n.a.	n.a.	n.a.
SL	0.24 ± 0.47	-0.31 ± 0.39	-0.51 ± 0.33	-0.67 ± 0.42	n.a.	n.a.	n.a.	n.a.
ST	-0.12 ± 0.39	-0.32 ± 0.35	-0.27 ± 0.30	-0.57 ± 0.45	n.a.	n.a.	n.a.	n.a.
AS	-0.13 ± 0.58	-0.16 ± 0.34	-0.16 ± 0.29	-0.31 ± 0.41	n.a.	n.a.	n.a.	n.a.
MZ	-0.28 ± 0.35	-0.34 ± 0.33	-0.23 ± 0.28	-0.36 ± 0.37	n.a.	n.a.	n.a.	n.a.
	**$\Delta(^{207}Pb/^{206}Pb)_{DGT\text{-}plant}$ / ‰**				**$\Delta(^{207}Pb/^{206}Pb)_{DGT\text{-}plant}$ / ‰**			
AS	0.14 ± 0.50	0.19 ± 0.38	0.12 ± 0.49	0.29 ± 0.38	-0.01 ± 0.28	-0.01 ± 0.28	-0.07 ± 0.42	0.10 ± 0.28
MZ	0.20 ± 0.59	0.24 ± 0.39	0.06 ± 0.53	0.35 ± 0.38	-0.20 ± 0.36	-0.20 ± 0.36	-0.38 ± 0.50	-0.09 ± 0.35
	**$\Delta(^{208}Pb/^{206}Pb)_{DGT\text{-}plant}$ / ‰**				**$\Delta(^{208}Pb/^{206}Pb)_{DGT\text{-}plant}$ / ‰**			
AS	0.21 ± 0.79	0.30 ± 0.41	0.28 ± 0.64	0.38 ± 0.43	0.09 ± 0.33	0.09 ± 0.33	0.08 ± 0.59	0.18 ± 0.34
MZ	0.15 ± 1.09	0.27 ± 0.51	0.17 ± 0.69	0.31 ± 0.49	-0.31 ± 0.44	-0.31 ± 0.44	-0.41 ± 0.64	-0.27 ± 0.41
	**$\Delta(^{206}Pb/^{204}Pb)_{DGT\text{-}plant}$ / ‰**				**$\Delta(^{206}Pb/^{204}Pb)_{DGT\text{-}plant}$ / ‰**			
AS	-0.12 ± 1.17	-0.13 ± 0.69	0.06 ± 0.76	-0.14 ± 0.69	0.22 ± 0.67	0.22 ± 0.67	0.41 ± 0.74	0.21 ± 0.66
MZ	-0.72 ± 1.56	-0.57 ± 1.04	-0.24 ± 1.08	-0.62 ± 0.98	-0.04 ± 0.73	-0.04 ± 0.73	0.29 ± 0.79	-0.09 ± 0.65

[a] Values are means ± U (k = 2). n.a. = not analyzed.

References

1. Wagner S, Santner J, Irrgeher J, Puschenreiter M, Happel S, Prohaska T. Selective Diffusive Gradients in Thin Films (DGT) for the Simultaneous Assessment of Labile Sr and Pb Concentrations and Isotope Ratios in Soils. Analytical Chemistry. 2022;94(16):6338-46. 10.1021/acs.analchem.2c00546.

2. National Institute of Standards and Technology (NIST). Certificate of Analysis Standard Reference Material® 987 Strontium Carbonate (Isotopic Standard) 2007. Available from: https://tsapps.nist.gov/srmext/certificates/987.pdf. Accessed on: 30.09.2025.

3. National Institute of Standards and Technology (NIST). Certificate of Analysis Standard Reference Material 981 Common Lead Isotopic Standard. 1991.

4. Retzmann A, Zimmermann T, Pröfrock D, Prohaska T, Irrgeher J. A fully automated simultaneous single-stage separation of Sr, Pb, and Nd using DGA Resin for the isotopic analysis of marine sediments. Analytical and Bioanalytical Chemistry. 2017;409(23):5463-80. 10.1007/s00216-017-0468-6.

5. Prohaska T, Watkins M, Latkoczy C, Wenzel WW, Stingeder G. Lead isotope ratio analysis by inductively coupled plasma sector field mass spectrometry (ICP-SMS) in soil digests of a depth profile. Journal of Analytical Atomic Spectrometry. 2000;15(4):365-9. 10.1039/A907203I.

6. Zimmermann T, Retzmann A, Schober M, Pröfrock D, Prohaska T, Irrgeher J. Matrix separation of Sr and Pb for isotopic ratio analysis of Ca-rich samples via an automated simultaneous separation procedure. Spectrochimica Acta Part B: Atomic Spectroscopy. 2019;151:54-64. https://doi.org/10.1016/j.sab.2018.11.009.

7. IUSS Working Group WRB. World Reference Base for Soil Resources 2014, update 2015: International soil classification system for naming soils and creating legends for soil maps. Rome: FAO; 2015. Available from: http://www.fao.org/3/a-i3794e.pdf. Accessed on: 19 January 2017.

8. Wageningen Evaluating Programs for Analytical Laboratories (WEPAL). Certificate of Analysis International Soil-Analytical Exchange REFERENCE MATERIAL ISE sample 885. 2020. Available from: https://participants.wepal.nl/webshop/quotation.php?action=download_certificate&ptprogram=Soil&file=/WEPAL/ISE-885.pdf. Accessed on: 29.09.2025.

9. National Institute of Standards and Technology (NIST). Standard Reference Material® 1547 Peach Leaves CERTIFICATE OF ANALYSIS. 2022. Available from: https://tsapps.nist.gov/srmext/certificates/archives/1547.pdf. Accessed on: 29.09.2025.

10. Wageningen Evaluating Programs for Analytical Laboratories (WEPAL). Certificate of Analysis International Plant-Analytical Exchange REFERENCE MATERIAL IPE sample 200. 2020. Available from: https://participants.wepal.nl/webshop/order.php?action=download_certificate&ptprogram=Plants&file=/WEPAL/IPE-200.pdf. Accessed on: 29.09.2025.

11. Linsinger T. Application Note 1 - Comparison of a measurement result with the certified value. Geel: European Commission - Joint Research Centre Institute for Reference Materials and Measurements (IRMM); 2010. Available from: https://ec.europa.eu/jrc/sites/default/files/erm_application_note_1_en.pdf. Accessed on: 08.10.2021.
